# The relative importance of kinetic mechanisms and variable enzyme abundances for the regulation of hepatic glucose metabolism – insights from mathematical modeling

**DOI:** 10.1186/s12915-016-0237-6

**Published:** 2016-03-02

**Authors:** Sascha Bulik, Hermann-Georg Holzhütter, Nikolaus Berndt

**Affiliations:** Charité – Universitätsmedizin Berlin, Institute of Biochemistry, Computational Systems Biochemistry Group, Charitéplatz 1, 10117 Berlin, Germany

**Keywords:** Diabetes, Enzyme abundance, Glucose metabolism, Hormonal enzyme regulation, Kinetic enzyme regulation, Liver, Reversible phosphorylation

## Abstract

**Background:**

Adaptation of the cellular metabolism to varying external conditions is brought about by regulated changes in the activity of enzymes and transporters. Hormone-dependent reversible enzyme phosphorylation and concentration changes of reactants and allosteric effectors are the major types of rapid kinetic enzyme regulation, whereas on longer time scales changes in protein abundance may also become operative. Here, we used a comprehensive mathematical model of the hepatic glucose metabolism of rat hepatocytes to decipher the relative importance of different regulatory modes and their mutual interdependencies in the hepatic control of plasma glucose homeostasis.

**Results:**

Model simulations reveal significant differences in the capability of liver metabolism to counteract variations of plasma glucose in different physiological settings (starvation, *ad libitum* nutrient supply, diabetes). Changes in enzyme abundances adjust the metabolic output to the anticipated physiological demand but may turn into a regulatory disadvantage if sudden unexpected changes of the external conditions occur. Allosteric and hormonal control of enzyme activities allow the liver to assume a broad range of metabolic states and may even fully reverse flux changes resulting from changes of enzyme abundances alone. Metabolic control analysis reveals that control of the hepatic glucose metabolism is mainly exerted by enzymes alone, which are differently controlled by alterations in enzyme abundance, reversible phosphorylation, and allosteric effects.

**Conclusion:**

In hepatic glucose metabolism, regulation of enzyme activities by changes of reactants, allosteric effects, and reversible phosphorylation is equally important as changes in protein abundance of key regulatory enzymes.

**Electronic supplementary material:**

The online version of this article (doi:10.1186/s12915-016-0237-6) contains supplementary material, which is available to authorized users.

## Background

An important feature of cellular metabolic networks is their ability to adjust the functional output to largely varying external conditions such as changes in nutrient supply, enforced synthesis of macromolecules during the growth phase, varying hormone levels, or presence of toxins. This adjustment is achieved by diverse regulatory mechanisms tuning the activities of enzymes and transporters in a concerted fashion. The simplest mechanism operative for virtually all reactions in the network consists in changes of the enzymatic turnover rate owing to concentration changes of substrates and products as long as these concentrations remain below the saturation level. Further, during the natural evolution of living systems, two basic regulatory mechanisms have been established to regulate the activity of enzymes and transporters in a metabolic network.

One such mechanism is to vary the amount of an enzyme, which linearly relates to the enzyme’s maximal catalytic capacity. Enzyme abundances can be regulated at the level of gene transcription, mRNA translation or protein degradation. In the rat liver, significant changes of enzyme amounts in the carbohydrate metabolism occur between 5 and 100 hours during a starvation-refeeding cycle [1, 2].

A second fundamental regulatory concept, usually termed kinetic regulation, consists of changes in the specific activity of enzymes due to conformational changes of the protein structure. This allows a rapid adaptation of the network to varying external and internal conditions within a few seconds. Conformational changes can be brought about by covalent or non-covalent binding of specific ligands (organic and inorganic metabolites, proteins, metal ions) or by partial proteolysis. The predominant concepts of kinetic regulation are allostery and reversible chemical modification. Allostery designates the modulation of enzyme kinetic properties by non-covalent binding of an effector molecule at the protein’s allosteric site, i.e. a site other than the binding site for the substrates and products. Reversible chemical modification encompasses the temporary covalent attachment of a molecule to and detachment from the enzyme protein. In a given metabolic system, not all enzymes are equally important for the regulation of the systemic behavior. Rather, only specific key enzymes carry the burden of the regulatory control.

High-throughput technologies enabling the cell-wide monitoring of RNA and protein levels have revealed high variability in the abundance of transcripts and protein amounts of metabolic enzymes, both among individual cells and among cells exposed to different physiological settings. This finding has promoted the current prevailing view that metabolic regulation is basically achieved by variable gene expression. For example, 90 % of contemporary scientific publications containing the keyword ‘metabolic regulation’ deal with variable gene expression of metabolic enzymes. This raises the question of whether metabolic regulation can really be inferred from changes in the expression profile of enzymes and transporters. Serious doubts in this simplifying concept emerge from several studies demonstrating that changes of pathway fluxes commonly displayed poor or even lacking correlation with changes of enzyme abundances [3, 4]. The need for inclusion of all modes of enzyme control in the regulation of cellular metabolism has already been pointed out by the time-dependent regulation analysis of Westerhoff et al. [5]. However, to date, such an analysis for a physiologically meaningful metabolic network is lacking. To this end, herein, we use a detailed kinetic model of hepatic glucose metabolism to quantify how variable protein abundances, changes of reactant concentrations, allosteric regulation, and reversible phosphorylation of metabolic enzymes contribute to the metabolic capacity of rat liver to assure the homeostasis of plasma glucose levels in different physiological settings including fasting, ad libitum feeding, and diabetes. The model takes into account allosteric effects as well as hormonal regulation of key metabolic enzymes by insulin and glucagon and thus allows a detailed recapitulation of kinetic regulation of the hepatic glucose metabolism. Experimentally determined enzyme abundances were used to scale the maximal rates of the respective enzymes. The model was parameterized and calibrated for rat hepatocytes because experimental information on uptake and release rates of glucose, enzyme-kinetic parameters, and condition-dependent differences in enzyme abundance is more extensive for rats than for humans.

In this work, we address two interrelated questions. First, what is the relative importance of different modes of enzyme regulation for the dynamic behavior of hepatic glucose metabolism? In other words, how would the regulation of the metabolic system change if one mode of regulation was lacking. Addressing this question we argue, from an evolutionary perspective, that owing to continuous mutational alterations of protein structures and natural selection, increasingly sophisticated mechanisms of enzyme regulation have been established. For example, allosteric regulation requires the enzyme protein to possess specific binding sites for potential effector molecules. Such binding sites will not have been present for primordial enzymes. A feasible computational method to study how the occurrence of different regulatory modes may have improved the regulation of a metabolic system is to compare the dynamic behavior of the system in the presence and absence of a specific regulatory mode. We performed this analysis by simulating the response of the net hepatic glucose uptake to varying external concentrations of plasma glucose by freezing those terms of the kinetic rate equations belonging to a given mode of regulation. The second question addressed in this work relates to the sensitivity of network fluxes against changes in the regulatory properties of individual enzymes. This is a question of practical importance for the design of drugs affecting the activity of selected target enzymes with the aim to change the dynamical behavior of the network into a desired direction. Here, it is important to know which regulatory mode of the target enzyme is best suited for such intervention. As the network response depends on the strength of the parameter change as well as on the external conditions, we restricted our analysis to selected stationary metabolic states of the liver and to small parameter changes allowing the application of sensitivity measures used in metabolic control theory.

## Methods

### Metabolic reactions

**Fig. 1:**
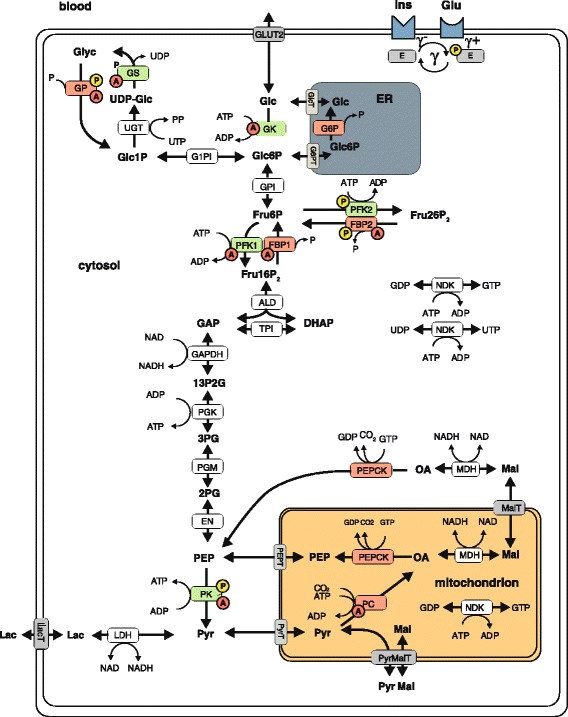
Schematic representation of the model of rat hepatocyte carbohydrate metabolism. The model contains enzymes involved in glycolysis, glyconeogenesis, and glycogen synthesis and utilization (ALD, EN, FBP1, FBP2, GAPDH, GK, GP, G6P, GPI, G1PI, GS, LDH, NDK, MDH, PC, PEPCK, PFK1, PFK2, PGK, PGM, PK, TPI, UGT) and transporters (ER < - > cytosol: GlcT, G6PT; mito < - > cytosol: MalT, PEPT, PyrMalT, PyrT; external space < - > cytosol: GLUT2, LacT). Enzymes (E) that are phosphorylated or dephosphorylated (γ) in response to insulin (Ins) and glucagon stimulus are marked by a yellow P, allosteric modification of enzymes is marked by a red A. The model contains the metabolites: DHAP, Fru6P, Fru16P_2_, Fru26P_2_, GAP, Glc, Glc1P, Glc6P, Glyc, Lac, Mal, OA, P, PEP, 13P2G, 2PG, 3PG, PP, Pyr, and UDP-Glc. The cofactors NAD, its reduced form NADH, ADP, and ATP are not treated as dynamic variables. GDP and GTP as well as UTP and UDP are generated from ATP and ADP by NDK. The physiological metabolic processes consuming Pyr in the hepatocyte during glycolysis are comprised into Lac formation and export. The rate equations are given in the Additional file 1. ADP, Adenosine diphosphate; ALD, Aldolase; EN, Enolase; ATP, Adenosine triphosphate; DHAP, Dihydroxyacetone phosphate; ER, Endoplasmic reticulum; FBP1, Fructose-1,6-bisphosphatase; FBP2, Fructose-2,6-bisphosphatase; Fru26P_2_, Fructose 2,6-bisphospate; GAP, Glyceraldehyde 3-phosphate; GAPDH, Glyceraldehyde 3-phosphate dehydrogenase; GDP, Guanosine diphosphate; GK, Glucokinase; GlcT, Glucose transporter; GLUT2, Glucose transporter 2; Glyc, Glycogen; G1PI, Glucose-1-phosphate isomerase; G6P, Glucose-6-phosphate phosphatase; GPI, Glucose-6-phosphate isomerase; GTP, Guanosine triphosphate; LacT, Lactate transporter; LDH, Lactate dehydrogenase; MDH, Malate dehydrogenase; mito, Mitochondrion; NAD, Nicotinamide adenine dinucleotide; NDK, Nucleoside-diphosphate kinase; P, Orthophosphate; PP, Pyrophosphate; Pyr, Pyruvate; PyrMalT, Pyruvate/malate antiporter; PC, Pyruvate carboxylase; PEPCK, Phosphoenolpyruvate carboxykinase; PEPT, Phosphoenolpyruvate transporter; PFK1/2, Phosphofructokinase-1/2; PGK, Phosphoglycerate kinase; PGM, Phosphoglycerate mutase; PK, Pyruvate kinase; PyrT, Pyruvate transporter; TPI, Triose-phosphate isomerase; UDP-Glc, uridine diphosphate glucose; UGT, Uridine diphospho-glucuronosyltransferase; UTP, Uridine triphosphate; 2PG, 2-phosphoglycerate; 3PG, 3-phosphoglycerate; 13P2G, 1,3-bisphosphoglycerate

**Fig. 2:**
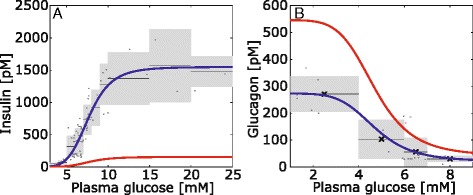
Glucose-hormone-transfer (GHT) functions. The GHT functions describe the dependence of plasma insulin (**a**) and plasma glucagon (**b**) on plasma glucose levels. Experimentally determined plasma concentrations of glucose and hormone (grey dots) from various sources (insulin: [59–61], glucagon: [42, 62–65]) were pooled (black lines – mean values, light grey boxes – standard deviations). The additional Tables S3 and S4 summarize the used values (Additional file 1). A Hill-type function was used to fit the data by least-square minimization yielding the normal GHT function (blue line). The red line depicts diabetic GHT function. For details of the used fit functions see Additional file 1

**Fig. 3:**
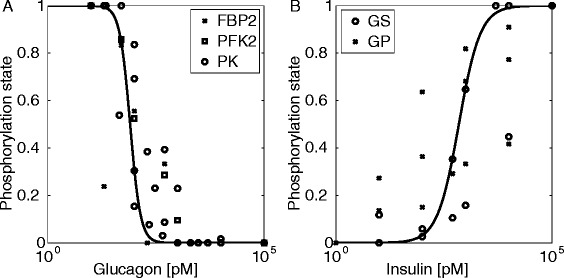
Phosphorylation states of enzymes as function of glucagon (**a**) or insulin (**b**) concentrations. Bold lines depict the function γ (Additional file 1) used to relate the level of insulin and glucagon to the phosphorylated form of enzymes regulated by reversible phosphorylation. Experimental data are from various sources [66–73]. Additional Tables S5 and S6 summarize the used values (Additional file 1)

The mathematical model of hepatic glucose metabolism encompasses the reactions of the pathways of glycolysis, gluconeogenesis, and glycogen turnover (Fig. 1). For all reactions, detailed liver-specific enzymatic rate equations have been established based on literature data (Additional file 1). Enzymatic rate equations describe the relationship between the reaction rate (i.e. amount of substrate converted into product per time unit) and the concentration of substrates, products, and allosteric effectors (activators or inhibitors). A list of fixed parameters is given in Additional Table S2 (Additional file 1). The overall rate law of enzymes regulated by reversible phosphorylation was composed as weighted linear combination of two rate laws, one holding for the phosphorylated form of the enzyme and the other holding for the dephosphorylated form. The weighting factor represents the relative fraction of the phosphorylated form and is related to the concentration of the hormones insulin and glucagon by empirical transfer and signal functions (Figs. 2 and 3, and Signaling transfer functions in Additional file 1). Parameterization of rate laws was carried out for rat hepatocytes.

**Fig. 4:**
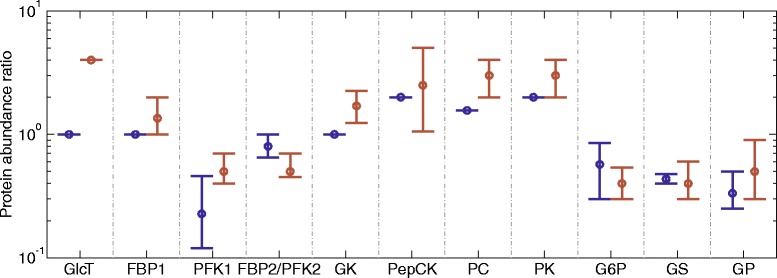
Experimentally determined variations in the abundance of key metabolic enzymes. The circles denote the average ratio between measured protein abundances in hepatocytes from fasted versus fed hepatocytes (blue) and diabetic versus normal hepatocytes (red). Vertical lines indicate the range of reported values. Experimental data are from various sources [2, 7, 19–38]

In all model simulations, we have chosen the exchange flux of glucose with the external space as the crucial target flux determining the capacity of the metabolic network to counteract changes in the plasma concentration of glucose. Numerical values for kinetic parameters of the enzymes were extrapolated from values determined in in vitro enzyme assays. However, numerical values for the maximal enzyme activities (V_max_) in vivo are unknown. The V_max_ values of enzymes depend on the (varying) expression level of the enzyme protein as well as effectors acting inside the cell but being (yet) unknown. Thus, there was no other option than to estimate these parameters by minimizing the difference between model predictions and experimental data. In order to use the model for predicting the impact of changes in the expression (= protein) level of enzymes elicited by changes in the physiological status (fed, fasted, normal, diabetic) of the rat it is compulsory to estimate the V_max_ values for only one of these states (serving as reference state) and to relate the V_max_ values for all other states to the V_max_ values of the reference state by the ratio of enzyme abundances, thereby exploiting the fact that the V_max_ value is a linear function of enzyme abundance. We have chosen the fed state as the reference case. With ${\mathit{\mathsf{V}}}_{\max}^{\mathit{\mathsf{fed}}}$ denoting the V_max_ value of an enzyme in the reference state, the V_max_ value of this enzyme in the fasted and diabetic state is put to${\mathit{\mathsf{V}}}_{\max}^{\mathit{\mathsf{f}\mathsf{asted}}}={\alpha}_{\mathit{\mathsf{f}\mathsf{asted}}}\;{\mathit{\mathsf{V}}}_{\max}^{\mathit{\mathsf{r}}\mathit{\mathsf{e}}\mathit{\mathsf{f}}}$ and ${\mathit{\mathsf{V}}}_{\max}^{\mathit{\mathsf{d}}}={\alpha}_{\mathit{\mathsf{d}}}\;{\mathit{\mathsf{V}}}_{\max}^{\mathit{\mathsf{r}}\mathit{\mathsf{e}}\mathit{\mathsf{f}}}$ where the scaling factors ${\alpha}_{\mathit{\mathsf{fasted}}}$ and ${\alpha}_{\mathit{\mathsf{d}}}$ are given by the ratio of mean enzyme abundances $\left\langle \mathit{\mathsf{E}}\right\rangle$ shown in Fig. 4:

$$ {\alpha}_{\mathit{\mathsf{fasted}}}=\raisebox{1ex}{${\left\langle \mathit{\mathsf{E}}\right\rangle}_{\mathit{\mathsf{fasted}}}$}\!\left/ \!\raisebox{-1ex}{${\left\langle \mathit{\mathsf{E}}\right\rangle}_{\mathit{\mathsf{fed}}}$}\right.\kern1em \mathit{\mathsf{and}}\kern1em {\alpha}_{\mathit{\mathsf{d}}}=\raisebox{1ex}{${\left\langle \mathit{\mathsf{E}}\right\rangle}_{\mathit{\mathsf{d}}}$}\!\left/ \!\raisebox{-1ex}{${\left\langle \mathit{\mathsf{E}}\right\rangle}_{\mathit{\mathsf{fed}}}$}\right. $$

We additionally defined a fourth physiological state, the so-called normal state, referring to a situation where the rat is kept under limited but energetically balanced food supply. As for the normal state no experimental data on enzyme abundances are available in the literature we made the assumption that the V_max_ values in the normal state can be reasonably well approximated by the V_max_ values in the fed and fasted state:

6 $$ {\alpha}_{\mathit{\mathsf{normal}}}=\frac{\mathsf{1}}{\mathsf{2}}\left({\alpha}_{\mathit{\mathsf{fasted}}}+{\alpha}_{\mathit{\mathsf{fed}}}\right) $$

With these settings, the only adjustable parameters to be estimated are the values of ${\mathit{\mathsf{V}}}_{\max}^{\mathit{\mathsf{fed}}}$ for the (fed) reference state.

### Relationship between plasma levels of glucose and the hormones insulin and glucagon

The phosphorylation state of enzymes controlled by reversible phosphorylation is determined by the insulin and glucagon concentrations within the liver sinusoids. Both hormones are secreted by the pancreas into the portal vein. The secretion rate is mainly controlled by the glucose concentration of the blood. An increase of glucose concentration stimulates the release of insulin from beta cells and reduces the release of glucagon from alpha cells in the pancreatic islets of Langerhans. Similarly to Koenig et al. [6], we established an empirical GHT function, which describes the relationship between the plasma level of glucose and of insulin and glucagon. To this end, we fitted a sigmoid function of Hill-type to a large data set of experimentally determined glucose-insulin and glucose-glucagon relations determined in the rat (Fig. 2).

### Relationship between plasma hormone level and phosphorylation state of enzymes

On the short-term, insulin and glucagon control the phosphorylation state of key regulatory enzymes by glucagon-stimulated enzyme phosphorylation and insulin-mediated inhibition of enzyme phosphorylation. We constructed an empirical signal function to describe the relationship between hormone levels and the relative share (γ) of the phosphorylated enzyme in the total enzyme pool (Fig. 3). We assumed that, at saturating concentrations of the hormone (set to 10^5^ pM), the phosphorylated fraction of the enzyme tends to γ = 1 (glucagon) or γ = 0 (insulin), respectively.

### Experimentally determined variations of enzyme abundances

Long-term alterations on the average values of plasma glucose and hormone concentrations induce changes in the abundance of key metabolic enzymes in the liver. Such adaptation occurs under extreme physiological and pathological settings like starvation or diabetes. Figure 4 shows the range of reported ratios of enzyme abundances, which were experimentally determined in fed and fasted hepatocytes and in ‘normal’ hepatocytes (for which the enzyme abundances were set to the mean values of abundances from fasted and fed hepatocytes) and diabetic hepatocytes. For example, the abundance of the glycolytic enzyme pyruvate kinase was found in different publications to be between two- and four-fold higher in diabetic hepatocytes compared with normal hepatocytes. We used the mean of the reported ranges for the fold-change of enzyme abundances depicted in Fig. 4 to scale the maximal enzyme activities when we parameterized the model for different physiological settings.

### Software

Computations were performed with MATLAB Release 2009a, The MathWorks, Inc., Natick, Massachusetts, United States. The SBML version of the model is supplied as Additional file 2.

## Results

### Validation of the model

We checked the validity of the kinetic model by comparing simulated glucose exchange fluxes, metabolite concentrations and filling states of the glycogen store with experimental data. Throughout the paper, we use the term glucose exchange flux to designate the glucose transport flux from the external space into the cell (= glucose net uptake), i.e. negative flux values indicate net release of glucose from the cell to the external space. The literature data used for the parametrization and validation of the model were obtained from liver tissue or hepatocytes of rats that have adopted different physiological states owing to either nutritional, genetic, or chemical interventions. The fed state designates a situation where rats are routinely fed ad libitum, i.e. food is available at all times in unlimited quantities. These animals are thus moderately obese and display elevated plasma glucose levels compared to rats in the normal state kept under well-controlled feeding. The fasted state is attained after 24 hours of complete food deprivation and is characterized by low plasma glucose levels. The diabetic state refers to a situation in which the animals exhibit an impaired glucose-insulin and glucose-glucagon relationship (for details see below). Finally, in the normal state, the rats have limited access to food without any signs of malnutrition. For the sake of convenience, livers or hepatocytes from fasted, fed, diabetic, and normal rats will be referred to as fasted, fed, diabetic, and normal livers/hepatocytes in the following sections.

The gene expression and, thus, the protein abundance of metabolic enzymes differs in these different physiological states. Figure 4 depicts ratios of enzyme abundances gathered from different literature sources. These ratios were used to put the maximal enzyme activities of different physiological states into a linear relation, thereby using the enzyme abundances in the fed state as reference. The relative maximal activities are given in Table 1. Using protein abundances for scaling maximal enzyme activities exploits the fact that the maximal activity of an enzyme is a linear function of the enzyme abundance with the turnover number (number of catalytic events per time unit) serving as a proportionality factor. For details of the procedure used to fix the numerical values of these scaling factors see Methods.

**Table 1:** Relative maximal enzyme activities for the normal, fasted, and diabetic rat liver. The numbers represent relative maximal enzyme activities with respect to those of the fed state (= reference state). They are based on the experimentally observed protein abundance ratios shown in Fig. 1. For the absolute values of maximal enzyme activities in the fed (reference) state see section model parameters in Additional file 1. Where there is no range given, only the given fixed ratio was used

Enzyme	Normal liver	Fasted liver		Diabetic liver	
	mean	mean	range	mean	range
FBP1	1.00	1.00	–	4.00	–
FBP2	0.66	0.43	0.4–0.475	0.26	0.20–0.40
GK	0.48	0.23	0.12–0.46	0.24	0.19–0.33
GlcT	1.00	1.00	–	1.35	1.00–2.00
GP	0.89	0.80	0.65–1.0	0.45	0.40–0.63
G6P	1.41	2.00	–	3.54	1.48–7.07
GS	1.00	1.00	–	1.70	1.24–2.25
PC	1.25	1.56	–	3.75	2.50–5.00
PEPCK	1.41	2.00	–	4.24	2.83–5.66
PFK1	0.76	0.57	0.3–0.85	0.30	0.23–0.41
PFK2	0.66	0.43	0.4–0.475	0.26	0.20–0.40
PK	0.58	0.33	0.25–0.5	0.29	0.17–0.52

*FBP1* Fructose-1,6-bisphosphatase, *FBP2* Fructose-2,6-bisphosphatase, *GK* Glucokinase, *GlcT* Glucose transporter, *G6P* Glucose-6-phosphate phosphatase, *GP* Glycogen phosphorylase, *GS* Glycogen synthase, *PC* Pyruvate carboxylase, *PEPCK* Phosphoenolpyruvate carboxykinase, *PFK1/2* Phosphofructokinase-1/2, *PK* Pyruvate kinase

#### Hepatic glucose production (HGP) from lactate

**Fig. 5:**
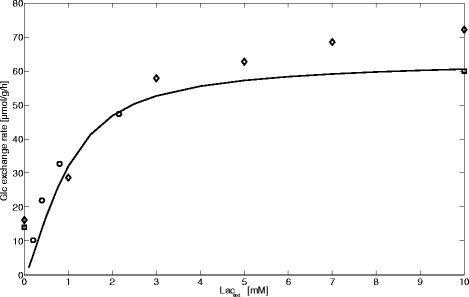
Gluconeogenesis from lactate. The solid line depicts the simulated stationary hepatic glucose exchange flux for fasted hepatocytes as function of the external lactate concentration (Lac_ext_), which was varied between 0–10 mM. Data points represent experimental data taken from [39–41] with different shapes for each experiment

In this simulation, we compared rates of gluconeogenesis (also referred to as hepatic glucose production, HGP) from lactate with experimental data obtained in perfused fasted livers (Fig. 5). We set the maximal enzyme activities to fasting conditions (Table 1) and varied the external concentration of lactate as done in various experiments. As the perfusion medium used in the experiments was devoid of glucose and hormones, we put these concentrations to zero. The integration time was chosen long enough to reach a stationary steady state. In agreement with the data, for lactate concentrations larger than 5 mM, the glucose exchange flux starts to become saturated to finally reach a maximum of approximately 60–70 μmol/g/L (= maximal net glucose production rate).

#### Transition between HGP and hepatic glucose utilization (HGU) in vivo

**Fig. 6:**
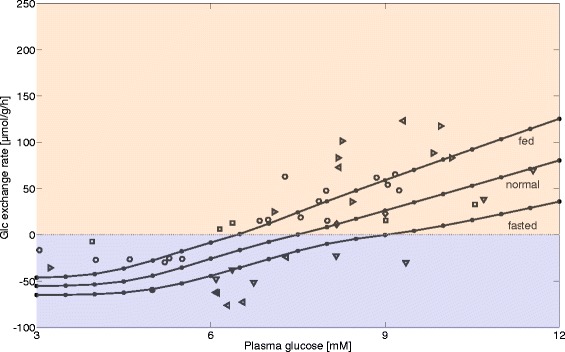
Glucose exchange flux in vivo in dependence of plasma glucose levels. The solid lines depict stationary hepatic glucose exchange rates for fasted, normal, and fed hepatocytes in dependence from the plasma glucose concentration. Data points represent experimental data taken from [6, 42–44] with different shapes for each experiment

In a second simulation, we varied the plasma glucose level between 3–12 mM and calculated the stationary glucose exchange flux of the fasted, normal, and fed liver in vivo. The relative enzyme abundances for the normal, fasted, and diabetic state relative to those of the fed (reference) state are given in Table 1. The plasma level of the hormones insulin and glucagon associated with a given value of plasma glucose was obtained by means of glucose-hormone transfer (GHT) functions (see Methods, Fig. 2). The external lactate concentration was put to the average physiological value of 1 mM. Again, we let the simulations run long enough to obtain a metabolic steady state. Figure 6 shows simulated and experimentally determined glucose exchange fluxes for normal, fasted, and fed livers in dependence of plasma glucose levels in vivo. Note that the experimental data in Fig. 6 merely indicate the expected range of glucose exchange fluxes because a clear assignment of these data to the specific state of the laboratory rat was not extractable from the literature sources. The simulated curves suggest that the glucose output of the liver is shifted to higher values in the fasted state compared with the fed state. In what follows, we define the glucose set point by the plasma glucose level at which the glucose exchange flux is zero, i.e. the liver neither utilizes nor produces glucose. This glucose set point is shifted from about 6.5 mM of the fed liver to about 9 mM of the fasted liver.

#### Hepatic glycogen storage during a starvation-refeeding cycle

**Fig. 7:**
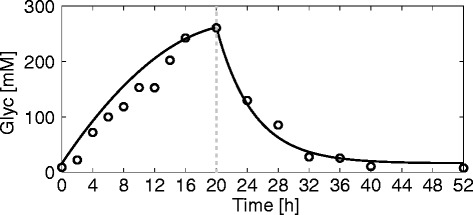
Hepatic glycogen storage. The solid line depicts the time course of intrahepatic glycogen content (Glyc) during refeeding and fasting of initially fasted hepatocytes. Open circles represent experimental data [45], where 48-h fasted rats were fed ad libitum for 20 h before they were fasted again. The broken vertical line indicates the transition from refeeding to fasting conditions

Thus far we considered stationary fluxes under various external conditions. Stationarity implies that the intrahepatic glycogen store also adopts a steady state where rates of glycogenolysis and glycogen synthesis are equal and thus do not contribute to the glucose exchange flux. However, in vivo, the plasma concentrations of glucose and hormones are continuously changing thus preventing the attainment of a steady state. Therefore, in a third simulation, we investigated the time-dependent variation of the glycogen pool during a starvation-refeeding cycle, which starts at time t = 0 with a fasted liver that is completely emptied in glycogen after 48 hours of starvation (plasma glucose concentration set to 4 mM). The corresponding glucagon and insulin concentrations were calculated from the GHT functions shown in Fig. 2. Figure 7 shows the simulated time-dependent filling state of the hepatic glycogen store together with experimental data during 20-h refeeding (plasma glucose concentration set to 8 mM) and subsequent fasting over a 32-h period. During the fasting period starting at t = 20 h, depletion of the glycogen store contributes to net glucose production.

#### Conversion of imported glucose to glycogen

**Fig. 8:**
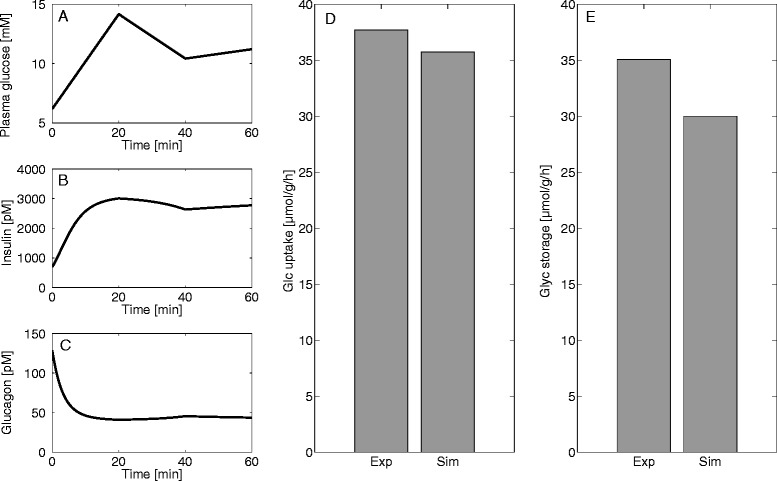
Glycogen production during the first hour of an oral glucose tolerance test. Experimental data (Exp) and the glucose profile (**a**) used as model input are taken from Niewoehner and Nuttall [6]. Insulin (**b**) and glucagon concentrations (**c**) were computed by means of the GHT function (Fig. 2). The bars depict the glucose exchange flux (= net glucose uptake) (**d**) and the cellular flux of glucose into the glycogen store (**e**) in units of glucose moieties for the first hour of an oral glucose tolerance test

In the feeding phase, hepatocytes take up plasma glucose that is either used to replenish their glycogen store or to form pyruvate via glycolysis. To check the relative share of these two alternative modes of glucose utilization we simulated an oral glucose tolerance test applied to fasted rats [7]. Figure 8 shows the average fluxes of glucose uptake and glycogen synthesis during the first 60 minutes. The absolute values of measured and computed flux rates as well as the flux ratios are in good concordance, indicating that more than 90 % of the glucose taken up by the fasted liver is used to replenish the glycogen store.

#### Range of intracellular metabolite levels

**Fig. 9:**
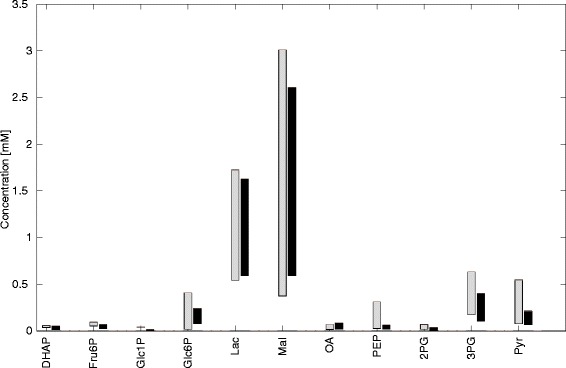
Simulated and measured concentration ranges of metabolites. Experimentally determined concentration ranges of metabolites (gray) are shown together with simulated concentration ranges (black) for the fed, normal, and fasted liver. Simulated concentration ranges were obtained as steady state concentrations when plasma glucose concentration was varied between 3–12 mM with constant plasma lactate (1 mM). Experimental data are from various experimental sources [46–54]. Experimental concentration values given in μmol/g wet weight were converted into mM by dividing these by the factor 0.46 and corrected for the liver density of 1.067 g/mL [55]. DHAP, Dihydroxyacetone phosphate; Fru6P, Fructose 6-phosphate; Glc1P, Glucose 1-phosphate; Glc6P, Glucose 6-phosphate; Mal, Malate; OA, Oxaloacetate; PEP, Phosphoenolpyruvate; 2PG, 2-Phosphoglycerate; 3PG, 3-Phosphoglycerate; Pyr, Pyruvate

Finally, we compared the intracellular metabolite concentrations in fed, normal, and fasted hepatocytes with reported tissue concentrations. To this end, we varied plasma glucose concentration within the physiological range of 3–12 mM and calculated the concentration range of all cellular metabolites occurring in the model. Lactate was fixed at 1 mM while glucagon and insulin concentrations were determined by means of the GHT functions (Fig. 2). Figure 9 demonstrates the good agreement between the computed ranges of intracellular metabolite concentrations for fasted and fed hepatocytes and ranges of reported experimental values.

### Model applications

#### The impact of variable protein abundance on glucose exchange fluxes in different conditions

The liver switches from glucose production to glucose utilization depending on the plasma glucose level and the two main hormones insulin and glucagon. Depending on the timing of nutrient uptake this switch may occur several times during a day and is mainly controlled by hormone-dependent reversible phosphorylation of key regulatory enzymes. If changes of the external conditions persist over several days or even longer time periods, regulation of protein abundance represents a further mechanism of metabolic adjustment. To reveal the physiological implications of metabolic adaptation through variable abundance of metabolic enzymes we calculated the glucose exchange flux of the liver over a broad range of blood plasma glucose concentrations. Lactate concentration was set to 1 mM, the intrahepatic filling state of the glycogen store was put to fixed values between 0 and 100 %, and insulin and glucagon values were determined by the GHT functions (Fig. 2).

**Fig. 10:**
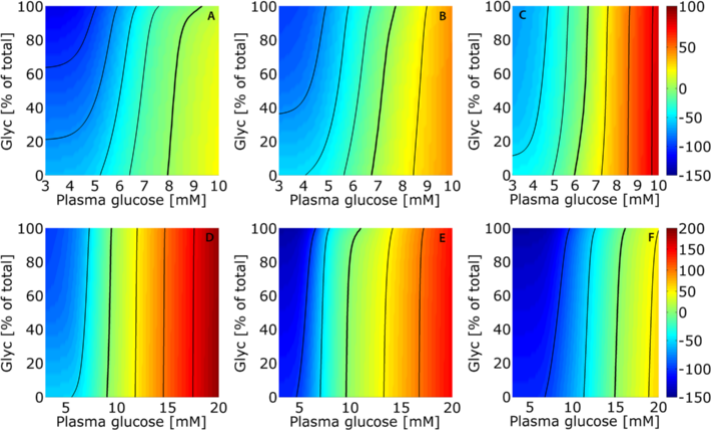
Stationary glucose exchange fluxes in dependence of plasma glucose and glycogen store. Plasma glucose was varied between 3 and 10 mM and filling state of glycogen storage was variably fixed to values between 0 and 100 %. The color encodes the steady state flux rates of glucose exchange of fasted (**a**), normal (**b**), fed (**c**), and diabetic hepatocytes (**d**–**f**). Green colors indicate small values of the glucose exchange flux around the set point where the net glucose exchange is zero (marked by bold black lines). Warm colors indicate net glucose uptake and cool colors indicate net glucose release. The legend is given on the right-hand axis in units of μmol/h/g tissue. Thin black isoclines connect equal exchange fluxes (in steps of 25 μmol/h/g tissue). Note that the set point values at 6.5 mM (fed), 7.5 mM (normal), and 9 mM (fasted) at half-filling of the glycogen store are identical with those in Fig. 6, where the glycogen contribution is zero due to the condition of stationarity. For the diabetic liver, the calculations were performed for three different scenarios: (**d**) no change of enzyme abundances compared with the normal state but impaired glucose-hormone relationship (see red curves of the GHT function in Fig. 2); (**e**) altered protein abundances (see Table 1 for scaling factors) but normal GHT function; and (**f**) combined effect of altered glucose-hormone relationship and protein abundances

Figure 10 depicts stationary exchange fluxes in response to varying plasma glucose levels at various filling states of the glycogen store. In the fasted liver, the glucose set point at which the glucose exchange flux is zero (indicated by bold black lines) lies between 8.0 and 9.2 mM depending on the filling state of the glycogen store. For the normal and fed liver, the set point is increasingly shifted to lower values lying between 6.7 and 7.7 mM and between 5.9 and 6.6 mM, respectively. These computations clearly demonstrate the impact of variable enzyme abundance on the capability of the liver to utilize or produce glucose at given plasma glucose levels – fasting shifts the range of glucose exchange rates into the direction of glucose production and decreases the capacity for glucose uptake. In contrast, fed hepatocytes possess about equal capacities for glucose production and glucose uptake.

Putting the filling state of the glycogen store to 100 % and the blood plasma glucose concentration to 3 mM and, conversely, putting the filling state of the glycogen store to zero and the blood plasma glucose concentration to 10 mM, the maximal observable rates of the glucose exchange flux predicted by the model were approximately between –70 μmol/g/h and +80 μmol/g/h for the fed hepatocyte and approximately between –120 μmol/g/h and +20 μmol/g/h for the fasted hepatocyte.

#### The impact of diabetes type 2 on the hepatic glucose exchange flux

The diabetic liver is not only characterized by changes in the abundance of metabolic enzymes (Table 1) but also by alterations in the glucose-hormone relationships (see GHT functions represented by the red curves in Fig. 2). Portal insulin was found to be reduced to about 10 %, while portal glucagon was increased to almost 200 % of normal concentrations [8]. In order to quantify the impact of either of these changes on the glucose exchange flux we performed three different simulations where changes in either the glucose-hormone response, the enzyme abundances, or both were taken into account.

We simulated the glucose exchange flux of the diabetic liver over a wide range of plasma glucose concentrations spanning from approximately 3 mM (observed during hypoglycemic crises) to approximately 20 mM (a commonly observed level in untreated diabetic rats [9]). Lactate concentration was set to 1 mM. Insulin and glucagon values were either computed by the normal or diabetic GHT (represented by the blue or red curves in Fig. 2, respectively).

Taking into account alterations of the glucose-hormone profiles only (Fig. 10d) our model predicts a shift of the set point to the right, i.e. an increase of the capability of the liver to function as glucose producer. This right-shift is even more pronounced if only changes in protein abundances are taken into account (Fig. 10e). The combined effect of altered hormonal control and altered enzyme abundances results in an additional right-shift of the set point such that the diabetic liver works as a glucose producer up to plasma glucose levels of 15 mM (Fig. 10f).

**Fig. 11:**
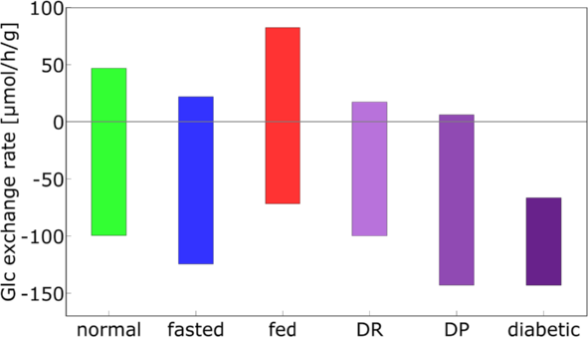
Maximal ranges of the glucose exchange fluxes. Plasma glucose was varied between 3 and 10 mM. Normal: protein abundance of normal hepatocytes; fasted: protein abundance of fasted hepatocytes; fed: protein abundance of fed hepatocytes; DR: diabetic GHT function, protein abundance of normal hepatocytes; DP: protein abundance of diabetic hepatocytes; diabetic: diabetic GHT function and protein abundance of diabetic hepatocytes

The importance of variable enzyme abundances for the adaptation of the liver to different physiological settings is summarized in Fig. 11 showing the calculated maximal range of glucose exchange rates at variations of the plasma glucose level between 3 and 10 mM, with and without adaptation of enzyme abundances and altered glucose-hormone relationship. These ranges were estimated by setting the filling of the glycogen store to 0 or 100 % at 3 mM and 10 mM plasma glucose, respectively.

#### Diurnal metabolic profiles of fasted, fed, and diabetic hepatocytes

We used the model to investigate the response of the liver to diurnal variations of the plasma glucose level in different physiological settings. Measured plasma glucose profiles monitored over 24 hours were used as model input. Since the diurnal plasma glucose levels differ significantly between the fasted, fed, and diabetic conditions, we used representative profiles for each condition. The associated hormone profiles were again calculated by means of the GHT function (Fig. 2). To estimate the impact of random individual variations in enzyme abundances on the simulation results we repeated the simulations 50 times with protein abundance ratios randomly sampled from the experimentally determined ranges (Table 1).

**Fig. 12:**
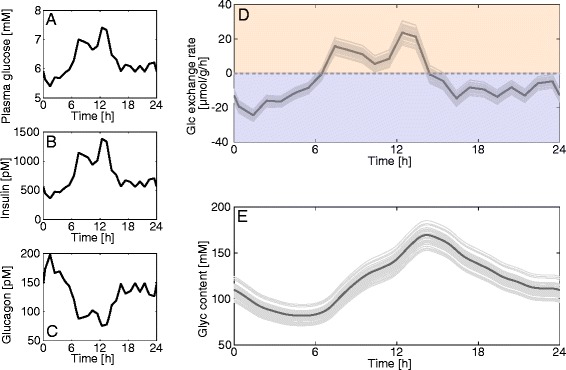
Diurnal variations of the glucose exchange flux and glycogen in the fed state. (**a**) Measured diurnal profiles of plasma glucose for fed hepatocytes taken from [56] and used as model input. (**b**, **c**) Diurnal profiles of insulin and glucagon calculated from the plasma glucose profile in (**a**) by means of the GHT function. (**d**) Simulated diurnal glucose exchange flux. (**e**) Simulated diurnal glycogen content in fed hepatocytes. The simulation was repeated 50 times with uniformly sampled protein abundances from the observed range for each enzyme (Table 1)

**Fig. 13:**
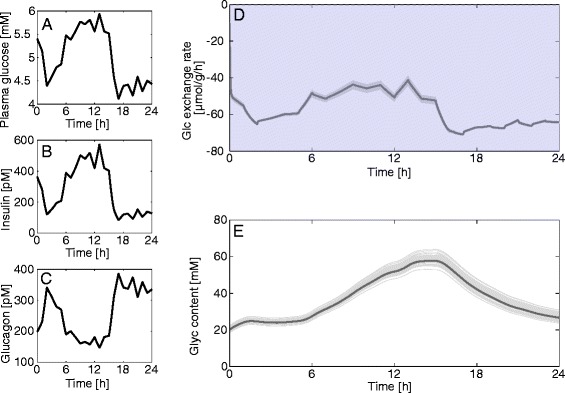
Diurnal variations of the glucose exchange flux and glycogen in the fasted state. (**a**) Measured diurnal profiles of plasma glucose for fasted hepatocytes taken from [56] and used as model input. (**b**, **c**) Diurnal profiles of insulin and glucagon calculated from the plasma glucose profile in (**a**) by means of the GHT function. (**d**) Simulated diurnal glucose exchange flux. (**e**) Simulated diurnal glycogen content in fasted hepatocytes. The simulation was repeated 50 times with uniformly sampled protein abundances from the observed range for each enzyme (Table 1)

Figures 12 and 13 show simulated diurnal variations of the glucose exchange flux for the fed and fasted state. At rich nutrient supply (fed state), the liver acts either as glucose producer or utilizer depending on the actual plasma glucose level (Fig. 12d). Over one day, glycogen decreases by approximately 50 %, but is fully replenished, resulting in no net glycogen utilization (Fig. 12e). Integrated over 1 day, the net glucose exchange rate of the liver is close to zero. In contrast, at fasting conditions, the model simulation predicts the liver to act persistently as glucose producer (Fig. 13d). Moreover, the hepatic glycogen store remains low over the whole day as it cannot be substantially replenished in phases of elevated plasma glucose (Fig. 13e).

**Fig. 14:**
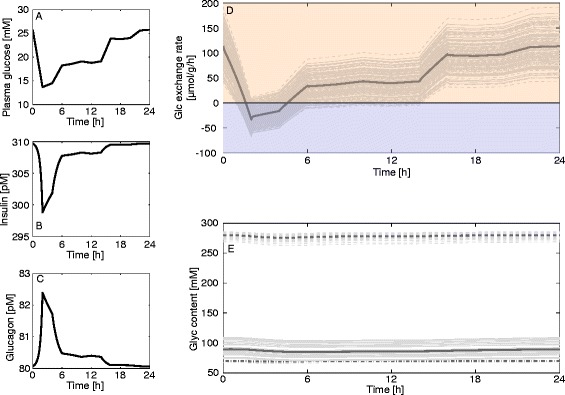
Diurnal variations of HGP/HGU and glycogen in the diabetic state. (**a**) Measured diurnal profiles of plasma glucose for diabetic hepatocytes taken from [8] and used as model input. (**b**, **c**) Diurnal profiles of insulin and glucagon calculated from the plasma glucose profile in (**a**) by means of the GHT function (Fig. 2, red curve). (**d**) Simulated diurnal glucose exchange flux. (**e**) Simulated diurnal glycogen content in diabetic hepatocytes. The simulation was repeated 50 times with uniformly sampled protein abundances from the observed range for each enzyme (Table 1). Due to conflicting experimental data regarding the amount of glycogen synthase (GS) and glycogen phosphorylase (GP) in diabetic hepatocytes, we set up three different scenarios: increased activity of GS by 70 % and diminished activity of GP by 50 % [29] (top trace – solid line); increased activity of GS by 70 % and decreased activity of GP by 50 % and reduced total glycogen storage capacity to 75 % [57] (bottom trace – dashed line); and decreased GS activity by 50 % and unchanged GP activity [58] (middle trace – dash-dotted line)

For the simulation of the diurnal glucose exchange flux of the diabetic liver (Fig. 14) we tested three different scenarios regarding the abundance of glycogen synthase and glycogen phosphorylase as the respective literature findings are controversial (see legend of Fig. 14). Here, glucose plasma levels are extraordinarily high, but insulin levels are still low due to impaired beta cell function, while glucagon levels are elevated. Although the plasma glucose remains persistently above 14 mM, there is a time window between 2 and 5 h where the liver acts as glucose producer. This is the result of the remarkable right-shift of the glucose set point (Fig. 10f). Glycogen levels range between almost filled and almost empty depending on enzyme abundance of glycogen synthase and glycogen phosphorylase.

The thin grey curves in Figs. 12, 13 and 14 illustrate how the diurnal profile of the glucose exchange flux is affected by variations of enzyme abundances around their mean. In these simulations, the enzyme abundances were randomly sampled within the reported ranges given in Table 1. The simulations reveal large differences in the impact of individual variations of enzyme abundances on the deviation of diurnal profile of the hepatic glucose exchange flux from the average. Whereas these deviations remain moderate for fed and fasted hepatocytes, they are substantially higher in diabetic hepatocytes because of large variations of enzyme abundances in this condition (see red bars in Fig. 4).

**Fig. 15:**
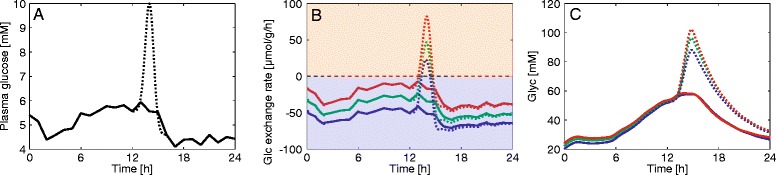
Different capabilities of fasted and fed hepatocytes to cope with transient hyperglycemic conditions. The figure depicts glucose exchange flux (**b**) and glycogen content (**c**) of fasted (blue), normal (green), and fed (red) hepatocytes in response to the 24-h glucose profile of fasted rats (**a**). The dotted lines refer to a situation where a transient glucose bolus (between 12 and 16 h) was added, driving the plasma glucose to a peak value of 10 mM. While the fasted hepatocyte has the highest glucose release rates in the unperturbed case it is clearly less efficient than the normal and fed hepatocyte to take up large amounts of glucose under sudden hyperglycemic conditions

Adaptation of the cellular enzyme endowment to long-term fed or fasted conditions shifts the metabolic output of the liver towards a better response to the ‘anticipated’ physiological state. However, this beneficial effect may turn to a regulatory disadvantage if the ‘anticipated’ external situation suddenly changes. This is illustrated in Fig. 15, showing the response of the glucose exchange flux to a perturbation of the typical plasma glucose profile for fasting conditions by a pulse-like increase of glucose as it may occur after intake of a glucose-rich meal. In the fasted state (liver metabolism shaped to deliver glucose to the plasma), the capability of the liver to rapidly and efficiently clear an excess of plasma glucose by increased glucose uptake and channeling into the glycogen pool is significantly lower than the capability of a liver which is adapted to persistent conditions of rich nutrient supply.

#### The impact of varying enzyme abundances, reversible phosphorylation and allosteric regulation on the long-term regulation of hepatic glucose metabolism

The central homeostatic function of the liver in the regulation of systemic glucose metabolism consists of efficiently counteracting deviations of plasma glucose from the normal level. This is reflected in the diurnal changes of the glucose exchange flux shown in Figs. 12, 13 and 14 mirroring, in an inverse manner, the variations of plasma glucose. Thus, a physiologically meaningful measure for the relative importance of various modes of metabolic regulation for plasma glucose homeostasis is the change of the diurnal profile of the glucose exchange flux that would result if changes of enzyme abundances, allosteric effects, and hormonal regulation were not present. As the reference state for such comparisons we have chosen the normal state, which refers to a situation where the rat is neither fasted nor fed ad libitum. Such a nutritional regime should better reflect the typical situation of a wild rat than extreme laboratory feeding regimes. The maximal enzyme capacities of the normal state are chosen as arithmetic mean of the maximal capacities in the fed and fasted state (Table 1). Technically, the absence of a specific regulatory mode was accomplished by freezing, in the kinetic rate equations, those terms belonging to a selected mode of regulation to their values adopted at the glucose set point of the normal state at which the glucose exchange flux is zero. Owing to this setting, the full model and the reduced models (lacking one mode of regulation) yield the same stationary state at the set point. Choosing the set point as the common point of reference for all model variants takes into account the fact that the homeostatic function of the liver with respect to plasma glucose consists in preventing larger deviations from the set point despite larger changes in plasma glucose.

For the comparison of the full model with the regulation-depleted models we used the following distance measure:

1 $$ \varDelta =\raisebox{1ex}{${\displaystyle {\ \int}_0^{24h}\left|{v}_{ex}^{normal}-{v}_{ex}^{\left(-\right)}\right|dt}$}\!\left/ \!\raisebox{-1ex}{${\displaystyle {\int}_0^{24h}\left|{v}_{ex}^{normal}\right|dt}$}\right. $$

**Fig. 16:**
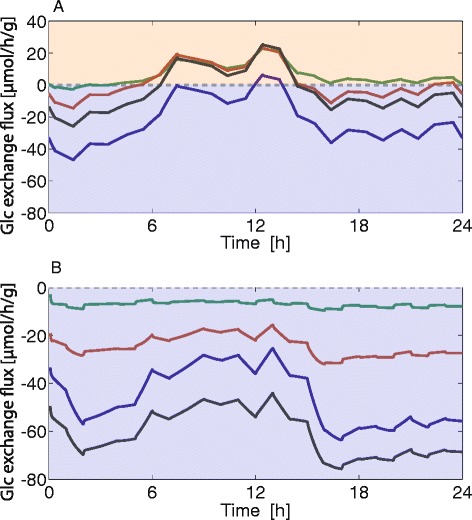
Influence of different levels of metabolic control on diurnal glucose exchange rates. Black curves: Full control – enzyme abundances are adapted to the fed (**a**) and fasted (**b**) state (Table 1) with full allosteric and hormonal control. Blue curves: No change of enzyme abundance – enzyme abundances of fed and fasted livers are the same as in the normal liver; full allosteric and hormonal control. Green curves: Lacking hormonal control – enzyme abundances are adapted to the fed (**a**) and fasted (**b**) state with full allosteric control. The value of the function γ controlling the ration between the phosphorylated and non-phosphorylated form of all enzymes is put to the constant value of 0.32, which holds at the reference case (= set point of the normal hepatocyte). Red curves: No allosteric regulation – enzyme abundances were adapted to the fed (**a**) and fasted (**b**) state, with full hormonal control. The saturation terms for allosteric effectors in the enzymatic rate equations were fixed to the values achieved in the reference state

Here, *v*_*ex*_^*normal*^ denotes the glucose exchange flux in the presence of all modes of metabolic regulation and *v*_*ex*_^(−)^ is the glucose exchange flux if one mode of regulation is dropped in the model simulations (for more details see legend of Fig. 16). From the values of Δ summarized in Table 2 and the corresponding diurnal profiles of the glucose exchange flux shown in Fig. 16 follows that all three modes of regulation have a significant impact on the response of the hepatic glucose exchange flux to variations of plasma glucose. Whereas the impact of the fast regulatory modes (reversible phosphorylation and allosteric regulation) is almost equal in the fed and fasted state, the change of enzyme abundances results in the essential mechanism in adapting the hepatic glucose metabolism to the fed state.

**Table 2:** Average curve difference Δ

Scenario	Difference of diurnal HGP/HGU profile	
	Fasted	Fed
No change of enzyme abundance	0.25	1.54
No allosteric effectors	0.60	0.47
No interconversion	0.89	0.88

#### Flux control coefficients, enzyme elasticities, and regulator strengths

##### Flux control coefficients

In the previous section, we studied the global impact of different modes of enzyme regulation on the metabolic response of the liver to varying plasma glucose concentrations. In this section, we use the model to study how individual enzymes are controlled by different modes of regulation and how they contribute to the overall regulation of the hepatic glucose exchange flux. The established method to address such questions is the Metabolic Control Analysis (MCA) [10]. In this concept, the regulatory importance of any reaction is quantified by its so-called flux control coefficient, defined as the relative change of the target flux of interest (in our case the glucose exchange flux, v_ex_) elicited by an infinitely small relative change in the flux (v_i_) of a single reaction:

2 $$ {\mathit{\mathsf{C}}}_{\mathit{\mathsf{i}}}=\frac{{\mathit{\mathsf{v}}}_{\mathit{\mathsf{i}}}}{{\mathit{\mathsf{v}}}_{\mathit{\mathsf{ex}}}}\frac{\partial {\mathit{\mathsf{v}}}_{\mathit{\mathsf{ex}}}}{\partial {\mathit{\mathsf{v}}}_{\mathit{\mathsf{i}}}} $$

According to the summation theorem, the flux control coefficients add up to unity. We calculated the control coefficients of the system for two extreme complementary physiological states, fasted hepatocytes at a hypoglycemic plasma glucose level of 4 mM and fed hepatocytes at a hyperglycemic plasma glucose level of 10 mM (Table 3).

**Table 3:** Control coefficients in the fasted and fed state. 0.00 means –0.005 < value <0.005

Enzyme	Fasted state		Fed state		Enzyme	Fasted state		Fed state	
	4 mM glucose ^a^	10 mM glucose ^b^	4 mM glucose ^b^	10 mM glucose ^a^		4 mM glucose ^a^	10 mM glucose ^b^	4 mM glucose ^b^	10 mM glucose ^a^
ALD	**0.00**	0.00	0.00	**0.00**	MDH_mito_	**0.00**	0.00	0.00	**0.00**
EN	**0.00**	0.00	0.00	**0.00**	NDK_UTP_	**0.00**	0.00	0.00	**0.00**
FBP1	**0.01**	–0.03	0.04	**0.00**	NDK_GTP_	**0.00**	0.00	0.00	**0.00**
FBP2	**0.00**	–2.01	0.12	**–0.18**	NDK_mito(GTP)_	**0.00**	0.00	0.00	**0.00**
GAPDH	**0.00**	0.00	0.00	**0.00**	PC	**0.66**	–0.25	0.60	**–0.01**
GK	**0.00**	2.87	–0.05	**0.56**	PEPCK	**0.00**	0.00	0.00	**0.00**
GlcT	**0.00**	0.27	0.04	**0.54**	PEPCK_mito_	**0.00**	0.00	0.00	**0.00**
GlcT_ER_	**0.00**	0.00	0.00	**0.00**	PEPT	**0.00**	0.00	0.00	**0.00**
GP	**–0.04**	0.11	–0.08	**0.00**	PFK1	**0.00**	0.22	0.00	**0.01**
G6P	**0.06**	–2.34	0.30	**–0.12**	PFK2	**0.00**	2.01	–0.12	**0.18**
GPI	**0.00**	0.00	0.00	**0.00**	PGK	**0.00**	0.00	0.00	**0.00**
G6PT_ER_	**0.00**	0.00	0.00	**0.00**	PGM	**0.00**	0.00	0.00	**0.00**
GS	**0.04**	–0.15	0.06	**0.00**	PK	**–0.01**	0.28	–0.06	**0.01**
LacT	**0.28**	0.01	0.14	**0.00**	PyrT	**0.00**	0.00	0.00	**0.00**
LDH	**0.00**	0.00	0.00	**0.00**	PyrMalT	**0.00**	0.00	0.00	**0.00**
MDH	**0.00**	0.00	0.00	**0.00**	TPI	**0.00**	0.00	0.00	**0.00**

For the numerical calculation of the control coefficients the amounts of the catalyzing enzymes were varied by 5 % (0.5 % yielded similar results). The sum of all control coefficients is equal to unity (summation theorem) within the limit of numerical accuracy

^a^ In bold font are the control coefficients for the physiological states (i.e. fasted protein profile and 4 mM plasma glucose and low glycogen store and fed protein profile and 10 mM blood glucose and high glycogen store)

^b^ In normal font are the control coefficients for the two states with the respective other protein profile. The reference flux values are –64 μmol/g/h for fasted hepatocytes and 81 μmol/g/h for fed hepatocytes

*ALD* Aldolase, *EN* Enolase, *ER* Endoplasmic reticulum, *FBP1* Fructose-1,6-bisphosphatase, *FBP2* Fructose-2,6-bisphosphatase, *GAPDH* Glyceraldehyde 3-phosphate dehydrogenase, *GK* Glucokinase, *GlcT* Glucose transporter, *G6P* Glucose-6-phosphate phosphatase, *GPI* Glucose-6-phosphate isomerase, *G6PT* Glucose-6-phosphate transporter, *GTP* Guanosine triphosphate, *GS* Glycogen synthetase, *LacT* Lactate transporter, *LDH* Lactate dehydrogenase, *MDH* Malate dehydrogenase, *mito* Mitochondrion, *NDK* Nucleoside-diphosphate kinase, *PyrMalT* Pyruvate/malate antiporter, *PC* Pyruvate carboxylase, *PEPCK* Phosphoenolpyruvate carboxykinase, *PEPT* Phosphoenolpyruvate transporter, *PFK1/2* Phosphofructokinase-1/2, *PGK* Phosphoglycerate kinase, *PGM* Phosphoglycerate mutase, *PK* Pyruvate kinase, *PyrT* Pyruvate transporter, *TPI* Triose-phosphate isomerase, *UTP* Uridine triphosphate

The control analysis revealed that the glucose exchange flux is under control of only seven enzymes (out of 32) exhibiting C_i_ values larger than 0.1 in at least one of the two physiological conditions studied (Table 3). Importantly, these key regulatory enzymes are known to change their abundance in response to altered external conditions. Moreover, each of these key regulatory enzymes is relevant in only one extreme physiological setting. In the fasted, hypoglycemic state, the glucose exchange flux is controlled by only two reactions catalyzed by pyruvate carboxylase and lactate transporter, which on the other hand exert no control in the fed, hyperglycemic state. Conversely, the hepatic glucose exchange flux in the fed, hyperglycemic state is controlled by fructose-2,6-bisphosphatase (FBP2), glucokinase (GK), glucose transporter, glucose-6-phosphate phosphatase, and phosphofructokinase-2 (PFK2), which exert no control in the complementary physiological setting.

**Fig. 17:**
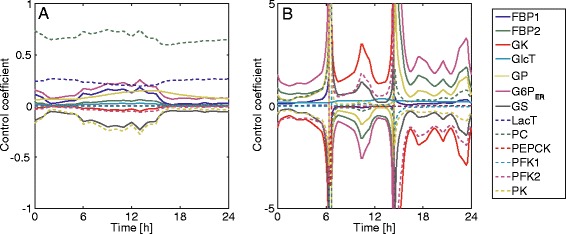
Control coefficients of regulatory enzymes. The control coefficients of the key regulatory enzymes are shown for the diurnal glucose profiles of the fasted (**a**) and fed (**b**) liver (see Figs. 12 and 13)

Figure 17 illustrates, for hepatocytes adapted to either fed or fasted conditions, the variation of the flux control coefficients of the relevant regulatory enzymes over one day. In the fed state, the variations are larger than in the fasted one and assume, in theory, infinitely large values at time points where the plasma glucose level approaches the set point at which the glucose exchange flux (appearing in the denominator of Equation 2) tends to zero during the switch from net glucose utilization to net glucose uptake and vice versa.

The flux control coefficient of a reaction is independent from the specific regulatory mechanism underlying the (small) change of the reaction rate. However, (small) relative changes of different kinetic parameters p_ik_ of the i-th enzyme may have largely different influence on the glucose exchange flux, which in the frame of MCA is expressed by the so-called response coefficient R_ik_, as follows:

3 $$ {\mathit{\mathsf{R}}}_{\mathit{\mathsf{i}}\mathit{\mathsf{k}}}=\frac{{\mathit{\mathsf{p}}}_{\mathit{\mathsf{i}}\mathit{\mathsf{k}}}}{{\mathit{\mathsf{v}}}_{\mathit{\mathsf{ex}}}}\frac{\partial {\mathit{\mathsf{v}}}_{\mathit{\mathsf{ex}}}}{\partial {\mathit{\mathsf{p}}}_{\mathit{\mathsf{i}}\mathit{\mathsf{k}}}}=\left[\frac{{\mathit{\mathsf{v}}}_{\mathit{\mathsf{i}}}}{{\mathit{\mathsf{v}}}_{\mathit{\mathsf{ex}}}}\frac{\partial {\mathit{\mathsf{v}}}_{\mathit{\mathsf{ex}}}}{\partial {\mathit{\mathsf{v}}}_{\mathit{\mathsf{i}}}}\right]\left[\frac{{\mathit{\mathsf{p}}}_{\mathit{\mathsf{i}}\mathit{\mathsf{k}}}}{{\mathit{\mathsf{v}}}_{\mathit{\mathsf{i}}}}\frac{\partial {\mathit{\mathsf{v}}}_{\mathit{\mathsf{i}}}}{\partial {\mathit{\mathsf{p}}}_{\mathit{\mathsf{i}}\mathit{\mathsf{k}}}}\right]={\mathit{\mathsf{C}}}_{\mathit{\mathsf{i}}}\;{\pi}_{\mathit{\mathsf{i}}\mathit{\mathsf{k}}} $$

According to Equation 3, the response coefficient R_ik_ is given as product of the flux control coefficient C_i_ and the so-called π-elasticity coefficient ${\pi}_{\mathsf{ik}}$

4 $$ {\pi}_{\mathit{\mathsf{i}}\mathit{\mathsf{k}}}=\frac{{\mathit{\mathsf{p}}}_{\mathit{\mathsf{k}}}}{{\mathit{\mathsf{v}}}_{\mathit{\mathsf{i}}}}\frac{\partial {\mathit{\mathsf{v}}}_{\mathit{\mathsf{i}}}}{\partial {\mathit{\mathsf{p}}}_{\mathit{\mathsf{k}}}} $$

quantifying the relative impact of the kinetic parameter p_ik_ on flux v_i_ [11]. Note that the π-elasticity coefficient ${\pi}_{\mathsf{ik}}$ has to be distinguished from the common elasticity coefficient

5 $$ {\varepsilon}_{\mathit{\mathsf{i}}\mathit{\mathsf{k}}}=\frac{{\mathit{\mathsf{E}}}_{\mathit{\mathsf{k}}}}{{\mathit{\mathsf{v}}}_{\mathit{\mathsf{i}}}}\frac{\partial {\mathit{\mathsf{v}}}_{\mathit{\mathsf{i}}}}{\partial {\mathit{\mathsf{E}}}_{\mathit{\mathsf{k}}}} $$

expressing the relative variation of the velocity v_i_ of the isolated enzyme caused by relative variations in the concentration of effector E_k_. Both coefficients are closely related because, in the rate equation, the dependence of the rate v_i_ from the effector E_k_ is usually a function of the term ${\mathit{\mathsf{X}}}_{\mathit{\mathsf{k}}}=\frac{{\mathit{\mathsf{E}}}_{\mathit{\mathsf{k}}}}{{\mathit{\mathsf{p}}}_{\mathit{\mathsf{k}}}}$ with p_k_ having the meaning of a binding constant of E_k_. In this case, it follows immediately from Equation 4 and Equation 5 that ${\varepsilon}_{\mathit{\mathsf{i}}\mathit{\mathsf{k}}}=-{\pi}_{\mathit{\mathsf{i}}\mathit{\mathsf{k}}}$, i.e. a large value of the π-elasticity coefficient implies a large value of the common elasticity coefficient and vice versa. We thus used π-elasticities to characterize the controllability of key regulatory enzymes by their effectors.

##### π-Elasticity coefficients

**Fig. 18:**
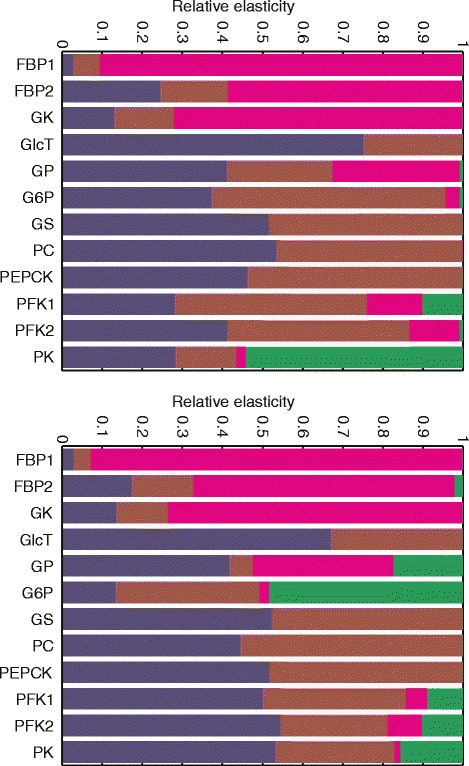
Relative enzyme π-elasticity coefficients. The π-elasticity coefficients (defined in Equation 4) with respect to protein abundance (blue), reactants (brown), allosteric effectors (pink), and reversible phosphorylation (green) for fasted hepatocytes at 4 mM plasma glucose (**a**) and fed hepatocytes at 10 mM plasma glucose (**b**). The elasticity coefficients for each enzyme were normalized to their absolute sum. For the reference flux values see legend of Table 3

In our model, the enzyme-kinetic parameters p_ik_ fall into four categories: (1) the maximal enzyme activity (V_max_) being a linear function of the enzyme abundance E_i_, (2) the binding constants for reactants (substrates and products), (3) the binding constants for allosteric effectors, and (4) the signal function γ determining the phosphorylation state of the enzyme and being itself a non-linear multi-parametric function of the plasma level of insulin and glucagon (see Additional file 1 and Methods, Fig. 3). By definition, the π-elasticity coefficient of an enzyme with respect to the enzyme’s abundance E_i_ is unity, i.e. the response coefficient with respect to E_i_ equals the flux control coefficient. For a better comparison of individual elasticity coefficients we calculated relative elasticity coefficients by relating the absolute value of an elasticity coefficient for a given enzyme to the sum of absolute values of all elasticity coefficients, i.e. the relative elasticity coefficients of an enzyme add up to unity. Figure 18 depicts the magnitude of the relative π-elasticity coefficients of the most important regulatory enzymes for the two complementary states, fasted hepatocytes at 4 mM plasma glucose and fed hepatocytes at 10 mM plasma glucose, considered in the previous sections. The complete list of elasticity coefficients is given in Additional Table S1 (Additional file 1).

Notably, with the exception of the glucose transporter, the kinetic effects caused by changes in the concentration of ligands and effectors prove to have a significant share in the total control of the key regulatory enzymes of hepatic glucose metabolism. For example, GK, which has a strong control of the glucose exchange flux in the fed state, exhibits a remarkable sensitivity against changes of its substrate glucose. GK activity is mainly regulated by a binding protein so that changes in the respective binding (Michaelis) constant have a large impact on the glucose exchange flux, especially at high plasma glucose levels. The physiological implications of the result of the control analysis are discussed below.

## Discussion

### General considerations

In this work, we used a detailed kinetic model of the hepatic glucose metabolism to investigate the contribution of the liver to the homeostasis of blood glucose under physiological and pathological conditions. We validated the model by comparing model simulations with a variety of experimental data on glucose exchange fluxes, metabolite concentrations and filling/emptying of the intrahepatic glycogen store. Our central goal was to dissect the relative importance of individual enzyme-regulatory mechanisms for the adequate response of the liver to varying plasma glucose levels. The general finding of this analysis is that changes in the hormone-induced phosphorylation state of key regulatory enzymes as well as changes in the concentration of allosteric effectors are at least of the same importance as changes in the enzyme abundance for the adjustment of the metabolic output to the metabolic demand defined by the external conditions. The strong influence of regulators beyond changes in protein abundance is presumably the main reason for the poor correlation usually observed between metabolic fluxes and abundance of the associated catalyzing enzymes.

The need for a concerted action of different modes of enzyme regulation can be reasoned by considering that even extremely high challenges to the adaptation of the cellular metabolism (e.g. during periods of starvation or overnutrition, exposure to toxic agents, inflammation or proliferation) are never constant over time but fluctuating, and thus cannot be met by just increasing or decreasing the abundance of enzymes. Therefore, it is fair to claim that any theoretical concept aiming at a better understanding of the regulation of metabolic networks has to take into account regulation of enzyme activities beyond the gene expression level.

### Functional consequences of changes in protein abundance during the transition between fasted and fed nutritional states and in diabetes

First, we analyzed how changes in the abundance of key metabolic enzymes reported for the rat liver under fasted and fed nutritional conditions influence the metabolic output at various physiological conditions. Changes in the abundance of key regulatory enzymes of the glycolytic and gluconeogenic pathways were found to entail significant differences in the relationship between plasma glucose levels and the hepatic glucose exchange fluxes – the ‘fasted’ liver becomes a stronger glucose producer, whereas the ‘fed’ liver becomes a stronger glucose utilizer. This adaptation is achieved by changes in enzyme abundance and is of advantage for the homeostasis of plasma glucose as long as the anticipated physiological situation persists. It may, however, turn to a disadvantage if a sudden (unexpected) change occurs. A liver adapted to fasting for 1–2 days is less prepared to respond to a sudden strong increase of plasma glucose than a liver experiencing continuously elevated plasma glucose levels (Fig. 15). A well-known clinical complication occurring during refeeding in strongly malnourished patients is glucose intolerance [12], a type of metabolic dysregulation that typically occurs in early stages of diabetes type 2. In line with this observation, the relation between plasma glucose levels and hepatic glucose exchange rates predicted by the model are very similar in fasting conditions and diabetes (Fig. 10), i.e. gluconeogenesis is increased and the glycolytic capacity reduced.

### Diurnal glucose production and utilization by the liver

The rates of HGP and HGU depend on the plasma level of nutrients and hormones that are permanently changing during the time course of the day. Taking measured diurnal plasma profiles as input, the kinetic model allows the simulation of diurnal changes of HGP and HGU and the filling state of glycogen (Figs. 12, 13 and 14). These simulations show that, in the fed nutritional state, the liver is able to switch between HGP and HGU. During the day time, the liver works predominantly as a glucose utilizer and during the night it is a glucose producer. Depending on the timing of food intake and the duration and intensity of physical exercise, the individual metabolic profiles may significantly differ from the generic profile used as input for our simulations. In contrast to the fed state, during long-term starvation, the liver is predicted to constantly work as a glucose producer to ensure that plasma glucose levels remain sufficiently high to fuel obligate glucose consumers such as the brain and erythrocytes. In the diabetic case, alterations in enzyme abundance of the glycolytic and gluconeogenetic enzymes together with impaired glucose hormone responses lead to a pathological shift towards gluconeogenesis (Fig. 11). The good concordance between the shift of the set point and the observed plasma glucose levels underpins the importance of the liver in determining plasma glucose levels. These fundamental differences in the basal metabolic states of the liver are also reflected in the filling states of glycogen. In the fed nutritional state, glycogen is degraded during HGP and replenished during HGU, glycogen store filling thereby varying between 50 % and 80 % of the total storage capacity. In the fasted state, the overall filling state of the glycogen store is much lower and varies between 10 % and 25 % only.

### Assessing the relative importance of variable enzyme abundance and kinetic regulation of enzyme activities for the regulation of hepatic glucose exchange rates

To investigate the relative importance of the different regulation modes of enzyme activities we simulated the glucose exchange flux of the liver for different nutritional states (Fig. 16 and Table 2). In the fed state, the strongest regulatory influence is exerted by the changes of enzyme abundances, whereas in the fasted state, reversible phosphorylation has the largest impact. An important finding is that the short-term metabolic adaptation of the liver can be largely attributed to hormonal regulation as the glucose exchange fluxes become almost constant when we fix the phosphorylation state of the interconvertible enzymes. Nevertheless, the impact of allosteric regulation is substantial both in the fasted and the fed state, accounting for approximately 50 % of flux changes brought about by reversible phosphorylation in the fasted and fed state, respectively. It has to be noted that the role of allosteric regulation is certainly underestimated in our model as the concentration values of important cofactors (adenosine tri-/di-/monophosphate, nicotinamide adenine dinucleotide, and its reduced form NADH) and of allosterically important metabolites of the citric acid cycle (e.g. citrate inhibition of the phosphofructokinase) were not taken into account.

### Metabolic control analysis (MCA)

To dissect the importance of individual enzymes for hepatic glucose exchange rates under different conditions we used the MCA concept. Calculation of flux control coefficients for the ‘fed’ and ‘fasted’ states revealed that enzymes carrying significant control are those showing significant changes of their abundance under different physiological conditions. Furthermore, the flux control is shared between different groups of enzymes in different conditions – enzymes being important in the glycolytic phase of liver metabolism are different from the ones central during gluconeogenesis (Table 3). Importantly, the control coefficients for the glucose exchange flux exhibit significant fluctuation over one day and diverge (by definition) when the glucose exchange flux is zero (Fig. 17).

We also calculated π-elasticity coefficients to quantify the relative share of reversible phosphorylation and concentration changes of reactants and allosteric effectors in the regulation of individual enzymes of hepatic glucose metabolism (Fig. 18). This analysis revealed a large variability in the relative contribution of the three fast regulatory modes to the control of regulatory enzymes and hence the control of glucose exchange flux.

Direct experimental validation of the computed elasticities in vivo is unfeasible because this would require monitoring of the glucose exchange flux of the liver at clamped plasma levels of glucose and hormones in response to the gradual variation of an effector specifically influencing one kinetic parameter of the target enzyme under study. As a surrogate, we checked whether the predicted elasticities are concordant with observed changes in plasma glucose levels induced by targeting a single key regulatory enzyme either by drugs or genetic interventions.

#### GK

The maximal control that can be exerted by GK is low in the fasted-hypoglycemic state but becomes large in the fed-hyperglycemic state. Experimentally, glucosamine-induced inhibition of GK caused only marginal reduction of glucose uptake in euglycemia, whereas in hyperglycemia a significant reduction of the net hepatic glucose uptake of about 40 % was observed [13], confirming our simulation results. Clinically, the regulatory importance of GK in hyperglycemia is used to target this enzyme in diabetes type 2 [14, 15].

#### Glycogen phosphorylase (GP)

Torres et al. [16] investigated the effects of a GP inhibitor (GP_I_) and metformin on hepatic glucose in presence of basal and four-fold increased levels of plasma glucagon in 18-h fasted conscious dogs. In euglycemic conditions, no change in the net hepatic glucose balance and plasma glucose was observed in the presence of GP_I_. However, after glucagon stimulation, the presence of GP_I_ significantly diminished the glucose output. Both findings confirm the predicted relatively high control of the GP in hypoglycemic conditions as well as the large share of allosteric regulation of this enzyme.

#### Phosphofructokinase-1 (PFK1)

To our knowledge, a rate-limiting role of this enzyme in the liver is not reported. In several non-hepatic tissues, PFK1 exerts only insignificant control of glycolytic flux [17], which agrees with the predicted very small values of control coefficients in both the hypo- and hyperglycemic cases.

#### PFK2/FBP2

The enzyme PFK2/FBP2 exerts control of the glucose exchange flux mainly by changes in its phosphorylation state, whereas changes in the abundance of this enzyme have no impact of the glucose exchange flux. The reason for the latter is the bifuncionality of this enzyme – the phosphorylated enzyme (PFK2) acts as a kinase catalyzing the formation of fructose 2,6-bisphosphate (Fru26P_2_), an efficient allosteric activator of PFK1, whereas the non-phosphorylated enzyme (FBP2) acts as a phosphatase catalyzing the degradation of Fru26P_2_ to fructose 6-phosphate (Fru6P). These two opposite reactions create a futile cycle Fru6P → Fru26P_2_ → Fru6P that consumes one molecule of ATP. Obviously, unequal modulation of these opposite activities cannot be achieved by changes of protein abundance because any change in enzyme amount influences both activities to the same extent and thus leaves the net flux unchanged. However, reversible phosphorylation enhances FBP2-activity and in parallel diminishes the PFK2-activity of this enzyme, resulting in a high sensitivity of the glucose exchange flux to changes in the phosphorylation state of the PFK2/FBP2.

#### Phosphoenolpyruvate carboxykinase (PEPCK)

Metabolic control of liver gluconeogenesis was quantified in groups of mice with varying PEPCK protein content. Surprisingly, livers with a 90 % reduction in PEPCK content showed only a 40 % reduction in gluconeogenic flux, indicating a lower than expected capacity for PEPCK protein content to control gluconeogenesis (estimated control coefficient of about 0.18) [18]. This is in good agreement with our theoretical predictions. She et al. [18] concluded that the liver PEPCK functions more as an integrator of hepatic energy metabolism than as a determinant of gluconeogenesis.

Knowledge of the flux control exerted by a specific enzyme and of the regulatory mechanisms that contribute to its control is valuable information for the design of new drugs. For example, our analysis revealed that changing the protein abundance of the bifunctional enzyme PFK2/FBP2 should have no influence on the stationary glucose exchange flux of hepatocytes. Hence, drugs targeting this enzyme as non-competitive inhibitors can be expected to have little impact on the modulation of the hepatic glucose exchange flux. However, our analysis suggests that drugs specifically targeting only the phosphorylated enzyme (phosphatase) or non-phosphorylated enzyme (kinase) have a strong impact on the glucose exchange flux. These theoretical findings are supported by the fact that cancer cells express a specific phosphatase (TIGAR) that catalyzes the degradation of the glycolytic activator Fru26P_2_ in order to suppress glycolysis and to redirect the glucose flux through the oxidative pentose phosphate pathway. Flux control by PFK2/FBP2 may serve as a good example of why up or down regulation of the abundance of an enzyme does not necessarily imply corresponding flux changes as is assumed in numerous publications dealing with the potential metabolic consequences of varying protein abundances.

## Conclusions

In summary, our work underlines the utility of kinetic modeling for the integration of experimental data from proteomics, metabolomics, and flux measurements, and for a wide range of physiological conditions into a unifying computational framework. Unraveling the role of different metabolic enzymes and different modes of enzyme regulation in the control of the hepatic glucose flux, the presented model may guide the design of novel drugs that reduce excessive glucose production of the liver in diabetic patients.

## Additional files

Additional file 1: Supplementary information for this publication is available in Additional file 1. (PDF 952 kb) Additional file 2: The SBML version of the model is supplied as Additional file 2. (XML 83 kb)

## Abbreviations

FBP2: Fructose-2,6-bisphosphatase
Fru26P_2_: Fructose 2,6-bisphosphate
Fru6P: Fructose 6-phosphate
GHT: Glucose hormone transfer
GK: Glucokinase
GP: Glycogen phosphorylase
GP_I_: Glycogen phosphorylase inhibitor
GPI: Glucose-6-phosphate isomerase
GS: Glycogen synthetase
HGP: Hepatic glucose production
HGU: Hepatic glucose utilization
MCA: Metabolic control analysis
PEPCK: Phosphoenolpyruvate carboxykinase
PEPT: Phosphoenolpyruvate transporter
PFK1/2: Phosphofructokinase-1/2
