# Supplementary material for: The relative importance of kinetic mechanisms and variable enzyme abundances for the regulation of hepatic glucose metabolism – insights from mathematical modeling
Source: BMC Biol. 2016 Mar 2;14:15. doi: 10.1186/s12915-016-0237-6 (PMC4774192; doi:10.1186/s12915-016-0237-6)
Supplement: Additional file 1: — Supplementary information for this publication is available in Additional file 1. (PDF 952 kb) [file 12915_2016_237_MOESM1_ESM.pdf]

Additional tables

Additional Table S1 Elasticities. 0.00 means -0.005 < value < 0.005.

Enzyme	Parameter	Fasted state		Fed state	
		**4 mM glucose**	10 mM glucose	4 mM glucose	**10 mM glucose**
FBP1	$k_i^{Fru26P_2}$	0,18	1,23	0,68	1,26
	K_i^{AMP}	2,20	2,47	2,24	2,49
	$k_m^{Fru16P_2}$	-0,69	-0,74	-0,59	-0,88
FBP2	k_i^{Fru6P}	0,77	0,82	0,80	0,84
	$k_m^{Fru26P_2}$	-0,64	-0,22	-0,27	-0,14
GlcT	k_m^{Glc}	0,14	0,26	0,15	0,27
	$k_m^{Glc_{ext}}$	0,19	0,25	0,17	0,22
GK	k_a^{Glc}	-3,26	-2,91	-3,19	-3,31
	f	-2,10	-1,77	-2,30	-1,87
	k_i^{Fru6P}	0,20	0,26	0,15	0,25
	k_m^{ATP}	-0,14	-0,14	-0,14	-0,14
	K_m^{Glc}	-1,01	-0,69	-1,03	-0,80
GP	K_a^{AMP}	0,09	0,15	0,07	0,19
	k_m^{Glyc}	-0,88	-1,30	-0,67	-1,60
	k_m^{P}	-0,69	-0,88	-0,52	-1,08
	K_m^{Glc1P}	0,00	0,00	0,00	0,00
	$K_{a_{Glc1P}}^{AMP}$	0,00	0,00	0,00	0,00
$G6P_{ER}$	$k_m^{G6P_{ER}}$	-0,94	-0,92	-0,93	-0,91
GS	$K_m^{UDP\text{-}Glc}$	0,85	0,44	1,12	0,35
	$K_0^{UDP\text{-}Glc}$	0,76	0,15	1,03	0,12

	K_a^{Glc6P}	0,49	0,13	0,75	0,11
	$K_b^{UDP\text{-}Glc}$	0,09	0,30	0,09	0,24
PC	$k_m^{ATP_{mito}}$	-0,01	-0,01	-0,01	-0,01
	$k_m^{Pyr_{mito}}$	-0,70	-0,58	-0,68	-0,48
	$k_m^{CO_{2mito}}$	-0,45	-0,45	-0,46	-0,45
PEPCK	k_m^{OA}	0,26	0,37	0,38	0,58
	k_m^{GTP}	0,43	0,51	0,54	0,65
	k_m^{PEP}	0,04	0,04	0,04	0,01
	k_m^{GDP}	-0,08	-0,06	0,02	0,00
	$k_m^{CO_2}$	-0,05	-0,04	0,04	0,00
PFK1	K_m^{ATP}	-0,05	-0,01	-0,04	0,00
	$K_m^{ATP}: K_a^{Fru26P_2}$	0,00	-0,01	-0,01	0,00
	K_i^{ATP}	4,37	3,96	4,29	3,97
	$K_i^{ATP}: f_{Fru26P_2}$	0,02	1,03	0,09	1,52
	$K_i^{ATP}: K_a^{Fru26P_2}$	-0,02	-0,97	-0,08	-1,39
	k_m^{Fru6P}	-2,30	-1,47	-2,19	-1,41
	$k_m^{Fru6P}: k_i^{ATP}$	2,17	1,25	2,04	1,21
	$k_m^{Fru6P}: k_i^{Cit}$	0,46	0,27	0,43	0,26
	$k_m^{Fru6P}: f_{AMP}$	3,33	1,87	3,10	1,81
	$k_m^{Fru6P}: K_a^{AMP}$	-1,52	-0,95	-1,44	-0,92
	$k_m^{Fru6P}: f_P$	9,84	4,97	9,15	4,85
	$k_m^{Fru6P}: K_a^{P}$	-0,87	-0,53	-0,82	-0,51
	$k_m^{Fru6P}: f_{Fru26P_2}$	0,09	6,41	0,52	10,09
	$k_m^{Fru6P}: K_a^{Fru26P_2}$	-0,09	-0,92	-0,46	-0,68
	$n^{Fru6P}: k_i^{ATP}$	0,02	0,01	0,01	0,00

	$n^{Fru6P}: f_{Cit}$	-0,68	-0,27	-0,52	-0,21
	$n^{Fru6P}: K_i^{Cit}$	0,01	0,00	0,01	0,00
	$n^{Fru6P}: f_{AMP}$	3,95	1,43	2,95	1,11
	$n^{Fru6P}: K_a^{AMP}$	-1,56	-0,63	-1,20	-0,49
	$n^{Fru6P}: f_P$	3,22	1,18	2,41	0,91
	$n^{Fru6P}: K_a^P$	0,00	0,00	0,00	0,00
	$n^{Fru6P}: f_{Fru26P_2}$	0,00	1,80	0,34	1,39
	$n^{Fru6P}: K_a^{Fru26P_2}$	0,00	0,00	-1,03	0,00
PFK2	k_m^{Fru6P}	-0,97	-0,43	-0,60	-0,42
	k_m^{ATP}	-0,13	-0,07	-0,13	-0,07
	n_0	-0,17	-0,16	-0,20	-0,09
	k_i^{PEP}	0,14	0,13	0,16	0,07
PK	k_m^{PEP}	-0,21	-0,21	-0,21	-0,23
	k_i^{ATP}	0,00	0,00	0,00	0,00
	$k_a^{Fru16P_2}$	-0,09	-0,08	-0,12	-0,03
	k_m^{ADP}	-0,33	-0,33	-0,33	-0,33

Additional Table S2 Fixed metabolite concentrations. Concentrations given in µmol/g wet weight were converted to mM by deviding with the factor 0.46 and corrected for the liver density of 1.067 g/ml [35].

**Metabolite**	**Value**	**Reference**
ADP	0.5 mM	[1]
ADP_{mito}	7.5 mM	[1, 2]
AMP	0.16 mM	[3, 4]
ATP	3.25 mM	[1]
ATP_{mito}	17.5 mM	[1, 2]

CO_2	5 mM	[5]
GDP + GTP	0.8 mM	[6, 7]
GDP_{mito} + GTP_{mito}	0.8 mM	[1, 2, 8]
NAD	1.13 mM	[9, 10]
NAD/NADH	1130	[11, 12]
NAD_{mito}	0.046 mM	[13]
$NAD_{mito}/NADH_{mito}$	11.5	[11, 12, 14]
P	5 mM	[3]
P_{mito}	8 mM	[15, 16]
PP	0.008 mM	[17]
UDP + UDP-Glc + UTP	1.2 mM	[3, 6, 7, 18, 19]
Vmm	-155 mV	[20]

Additional Table S3 Data for the GHT functions – insulin (Figure 18A).

Glc [mM]	Insulin [pM]	Reference
5.92	490.2	[21]
6.36	524.6	
6.37	656.2	
6.46	627.2	
6.48	489.1	
6.66	627.2	
6.77	592.8	
6.80	619.5	
6.80	613.7	
6.88	541.5	

6.91	481.8	
6.91	388.7	
7.01	560.9	
7.02	415.8	
7.34	652.5	
7.34	432.7	
7.41	592.8	
7.41	751.8	
7.42	634.5	
7.42	913.0	
7.44	634.2	
7.44	564.6	
7.52	605.2	
7.62	817.7	
7.97	766.1	
8.16	722.5	
9.13	1337.9	
9.47	818.8	
10.01	835.7	
10.19	1384.1	
14.67	1433.2	
18.33	1170.5	
20.47	1180.0	
24.09	1440.2	
25.45	1538.0	

25.79	1748.3	
5.20	607.7	[22]
5.60	448.6	
5.90	448.6	
5.90	448.6	
5.90	448.6	
5.90	448.6	
5.90	390.7	
6.20	549.8	
6.20	405.1	
6.60	752.4	
7.60	1056.3	
7.70	1215.4	
7.70	1099.7	
8.70	1114.1	
9.40	1461.4	
9.80	1504.8	
12.60	1823.2	
15.70	1967.8	
3.86	30.2	[23]
3.96	61.8	
3.96	59.3	
3.98	47.0	
4.04	50.3	
4.18	30.2	

4.36	84.0	
4.55	57.9	
4.63	121.1	
5.05	113.2	
5.18	155.7	
5.49	113.2	
5.77	168.1	
5.77	200.2	
5.79	197.7	
5.83	242.2	
5.92	151.0	
6.01	197.7	
6.05	207.6	
6.20	205.1	
6.22	185.4	
6.32	171.1	
6.46	168.6	
6.63	244.1	
6.71	198.8	
6.73	181.2	
6.75	151.0	
6.79	158.5	

Additional Table S4 Data for the GHT functions – glucagon (Figure 18B).

Glc [mM]	Glucagon [pM]	Reference
6.17	30.18	[24]
6.19	30.18	
6.28	30.65	
6.45	33.79	
6.60	43.54	
6.62	34.73	
6.65	44.17	
6.73	46.52	
6.92	27.50	
7.28	35.68	
5.31	56.41	[25]
5.93	32.32	
6.00	28.30	
6.35	29.37	
8.18	31.25	
8.70	26.70	
1.69	368.32	[26]
7.68	9.78	
1.93	210.74	[27]
2.22	324.83	
7.78	36.73	
7.78	40.01	

1.30	254.93	[28]
1.30	268.79	
2.24	199.49	
5.08	136.62	
5.97	188.60	
6.05	163.36	
6.27	185.63	

Additional Table S5 Data for the phosphorylation states of enzymes as function of glucagon concentrations (Figure 19A).

Enzyme	Glucagon [pM]	Relative activity	Reference
FBP2	1.00E-03	1.00	[29]
	1.00E+01	1.00	
	5.00E+01	0.83	
	1.00E+02	0.56	
	5.00E+02	0.33	
	1.00E+03	0.00	
	1.00E+05	0.00	
	2.00E-03	1.00	[30]
	2.00E+01	0.24	
	2.00E+02	0.00	
	2.00E+03	0.00	
PFK2	1.00E-03	1.00	[29]
	1.00E+01	1.00	
	5.00E+01	0.86	
	1.00E+02	0.52	

	5.00E+02	0.29	
	1.00E+03	0.10	
	1.00E+05	0.00	
PK	1.00E-03	1.00	[29]
	1.00E+01	1.00	
	5.00E+01	1.00	
	1.00E+02	0.30	
	5.00E+02	0.09	
	1.00E+03	0.00	
	1.00E+05	0.00	
	1.00E-02	1.00	[31]
	1.00E+02	0.69	
	3.00E+02	0.23	
	1.00E+03	0.00	
	3.00E+03	0.00	
	1.00E+04	0.00	
	1.00E+01	1.00	[32]
	2.20E+01	1.00	
	4.50E+01	0.54	
	1.00E+02	0.15	
	2.20E+02	0.08	
	4.50E+02	0.03	
	1.00E+03	0.00	
	1.00E-03	1.00	[33]
	1.00E+02	0.84	

	5.00E+02	0.39	
	1.00E+03	0.23	
	5.00E+03	0.00	
	1.00E+04	0.02	
	2.00E-03	1.00	[30]
	2.00E+01	1.00	
	2.00E+02	0.38	
	2.00E+03	0.00	

Additional Table S6 Data for the phosphorylation states of enzymes as function of insulin concentrations (Figure 19B).

Enzyme	Insulin[pM]	rel. Activity	Reference
GP	1.00E+00	0.00	[34]
	1.00E+02	0.15	
	5.00E+02	0.29	
	1.00E+03	0.33	
	1.00E+04	0.42	
	1.00E+05	1.00	
	1.00E-03	0.00	[35]
	1.00E+01	0.14/0.27	
	1.00E+02	0.36/0.64	
	1.00E+03	0.68/0.82	
	1.00E+04	0.77/0.91	
	1.00E+05	1.00	
GS	1.00E+01	0.00	[34]
	1.00E+02	0.03	

	5.00E+02	0.11	
	1.00E+03	0.16	
	1.00E+04	0.45	
	1.00E+05	1.00	
	1.00E-03	0.18	[36]
	1.00E+00	0.00	
	1.00E+01	0.12	
	1.00E+02	0.06	
	5.00E+02	0.35	
	1.00E+03	0.65	
	5.00E+03	1.00	
	1.00E+04	1.00	

Signaling transfer functions

Hormone dependency on glucose

$$\mathrm{Ins} = 1.55\ \mathrm{nM} * \frac{(\mathrm{Glc_{ext}})^{5.7}}{(\mathrm{Glc_{ext}})^{5.7} + (7.7\ \mathrm{mM})^{5.7}}$$

$$\mathrm{Glucagon} = 0.253\ \mathrm{nM} * \left(1 - \frac{(\mathrm{Glc_{ext}})^{5.65}}{(\mathrm{Glc_{ext}})^{5.65} + (4.7\ \mathrm{mM})^{5.65}}\right) + 0.02\ \mathrm{nM}$$

$$\mathrm{Ins_{diab}} = 0.155\ \mathrm{nM} * \frac{(\mathrm{Glc_{ext}})^{5.7}}{(\mathrm{Glc_{ext}})^{5.7} + (7.7\ \mathrm{mM})^{5.7}}$$

$$\mathrm{Glucagon_{diab}} = 0.506\ \mathrm{nM} * \left(1 - \frac{(\mathrm{Glc_{ext}})^{5.65}}{(\mathrm{Glc_{ext}})^{5.65} + (4.7\ \mathrm{mM})^{5.65}}\right) + 0.04\ \mathrm{nM}$$

Transfer function of external hormones to phosphorylation state

$$\gamma = \frac{1}{2} * \left(1 - \frac{Ins^{1.75}}{Ins^{1.75} + (0.70\ nM)^{1.75}} + \frac{Glucagon^{3.6}}{Glucagon^{3.6} + (0.08\ nM)^{3.6}}\right)$$

Stoichiometric matrix

$$\frac{d}{dt}DHAP = v_{ALD} - v_{TPI}$$

$$\frac{d}{dt}Fru16P_2 = v_{PFK1} - v_{FBP1} - v_{ALD}$$

$$\frac{d}{dt}Fru26P_2 = v_{PFK2} - v_{FBP2}$$

$$\frac{d}{dt}Fru6P = v_{GPI} - v_{PFK1} - v_{PFK2} + v_{FBP1} + v_{FBP2}$$

$$\frac{d}{dt}GAP = v_{ALD} + v_{TPI} - v_{GAPDH}$$

$$\frac{d}{dt}GDP = -v_{NDK^{GTP}} + v_{PEPCK}$$

$$\frac{d}{dt}GDP_{mito} = -v_{NDK^{GTP_{mito}}} + v_{PEPCK_{mito}}$$

$$\frac{d}{dt}Glc = v_{GLUT2} - v_{GK} + v_{GlcT_{ER}}$$

$$\frac{d}{dt}Glc_{ER} = v_{G6P_{ER}} - v_{GlcT_{ER}}$$

$$\frac{d}{dt}Glc1P = v_{GP} - v_{G1PI} - v_{UGT}$$

$$\frac{d}{dt}Glc6P = v_{GK} + v_{G6PT_{ER}} - v_{GPI} + v_{G1PI}$$

$$\frac{d}{dt}Glc6P_{ER} = -v_{G6P_{ER}} - v_{Glc6PT_{ER}}$$

$$\frac{d}{dt}Glyc = v_{GS} - v_{GP}$$

$$\frac{d}{dt}GTP = v_{NDK^{GTP}} - v_{PEPCK}$$

$$\frac{d}{dt}GTP_{mito} = v_{NDK^{GTP_{mito}}} - v_{PEPCK_{mito}}$$

$$\frac{d}{dt}Lac = v_{LacT} + v_{LDH}$$

$$\frac{d}{dt}Mal = v_{MalT} - v_{MDH} + v_{PyrMalT}$$

$$\frac{d}{dt}Mal_{mito} = -v_{MalT} - v_{MDH_{mito}} - v_{PyrMalT}$$

$$\frac{d}{dt}OA = v_{MDH} - v_{PEPCK}$$

$$\frac{d}{dt}OA_{mito} = v_{PC} - v_{PEPCK_{mito}} + v_{MDH\,mito}$$

$$\frac{d}{dt}PEP = v_{EN} - v_{PK} + v_{PEPCK} - v_{PEPT}$$

$$\frac{d}{dt}PEP_{mito} = v_{PEPCK_{mito}} + v_{PEPT}$$

$$\frac{d}{dt}13P2G = v_{GAPDH} - v_{PGK}$$

$$\frac{d}{dt}2PG = v_{PGM} - v_{EN}$$

$$\frac{d}{dt}3PG = v_{PGK} - v_{PGM}$$

$$\frac{d}{dt}Pyr = v_{PK} - v_{LDH} - v_{PyrT} - v_{PyrMalT}$$

$$\frac{d}{dt}Pyr_{mito} = v_{PyrT} - v_{PC} + v_{PyrMalT}$$

$$\frac{d}{dt}UDP = -v_{NDK^{UTP}} + v_{GS}$$

$$\frac{d}{dt}UDP\text{-}Glc = v_{UGT} - v_{GS}$$

$$\frac{d}{dt}UTP = v_{NDK^{UTP}} - v_{UGT}$$

Reaction kinetics

ALD (Aldolase)

$$Fru16P_2 \leftrightarrow GAP + DHAP$$

$$v_{ALD} = v_{max}^{ALD} \cdot \frac{Fru16P_2 - GAP \cdot DHAP/k_{eq}^{ALD}}{\left(1 + \frac{Fru16P_2}{k_m^{Fru16P_2}}\right) + \left(1 + \frac{GAP}{k_m^{GAP}}\right)\left(1 + \frac{DHAP}{k_m^{DHAP}}\right) - 1}$$

$$v_{max}^{ALD} = 7.78 \cdot 10^8\ h^{-1}$$

k_{eq}^{ALD} = 0.099 mM [37]

$k_m^{Fru16P_2}$ = 0.004 mM [38]

k_m^{GAP} =0.48 mM [39]

K_m^{DHAP} = 0.38 mM [39]

EN (Enolase)

$2PG \leftrightarrow PEP$

$$v_{EN} = v_{max}^{EN} \cdot \frac{2PG - PEP/k_{eq}^{EN}}{1 + \frac{2PG}{k_m^{2PG}} + \frac{PEP}{k_m^{PEP}}}$$

$v_{max}^{EN} = 1.94 \cdot 10^{10} h^{-1}$

k_{eq}^{EN} = 1.7 [40]

$k_m^{2PG} = 0.14\, mM$ [41]

$k_m^{PEP} = 0.31\, mM$ [41]

FBP1 (Fructose-1,6-bisphosphatase)

$Fru16P_2 \rightarrow Fru6P + P$

$$v_{FBP1} = V_{max}^{FBP1} * \left((1 - \gamma^{FBP1}) * v_{FBP1}^{native} + \gamma^{FBP1} * v_{FBP1}^{phospho}\right)$$

$V_{max}^{FBP1} = 2.92 \cdot 10^4 mM \cdot h^{-1}$

$$v_{FBP1}^{native} = \frac{Fru16P_2}{Fru16P_2 + k_{m^{native}}^{Fru16P_2}} / \left(1 + \frac{Fru26P_2^n}{(k_i^{Fru26P_2})^n}\right) / \left(1 + \left(\frac{AMP}{K_i^{AMP}}\right)^{n_{AMP}}\right)$$

$k_{m^{native}}^{Fru16P_2}$ = 0.0029mM [42]

$k_i^{Fru26P_2}$ = 0.00113 mM [42]

n = 1.26 [42]

n_{AMP} = 2.43 [42]

K_i^{AMP} = 0.023 mM [42]

$$v_{FBP1}^{phospho} = \frac{Fru16P_2}{Fru16P_2 + k_{m^{phospho}}^{Fru16P_2}} / \left(1 + \frac{Fru26P_2^n}{(k_i^{Fru26P_2})^n}\right) / \left(1 + \left(\frac{AMP}{K_i^{AMP}}\right)^{n_{AMP}}\right)$$

$k_{m^{phospho}}^{Fru16P_2} = 0.0019$ mM [42]

$k_i^{Fru26P_2} = 0.00113$ mM [42]

$n = 1.26$ [42]

$K_i^{AMP} = 0.023$ mM [42]

$n_{AMP} = 2.43$ [42]

GAPDH (Glyceraldehyde 3-phosphate dehydrogenase)

$$GAP + P + NAD^+ \leftrightarrow 13P2G + NADH + H^+$$

$$v_{GAPDH} = v_{max}^{GAPDH} * \frac{NAD^+ \cdot GAP \cdot P - 13P2G \cdot NADH / k_{eq}^{GAPDH}}{\left(1 + \frac{NAD^+}{k_m^{NAD^+}}\right) \cdot \left(1 + \frac{GAP}{k_m^{GAP}}\right) \cdot \left(1 + \frac{P}{k_m^P}\right) + \left(1 + \frac{NADH}{k_m^{NADH}}\right) \cdot \left(1 + \frac{13P2G}{k_m^{13P2G}}\right) - 1}$$

$v_{max}^{GAPDH} = 2.92 \cdot 10^8 h^{-1} \cdot mM^{-2}$

$k_{eq}^{GAPDH} = 10^{-4} mM^{-1}$ [43]

$k_m^{NAD^+} = 0.010$ mM [44]

$k_m^{GAP} = 0.035$ mM [44]

$k_m^P = 3.8$ mM [45]

$k_m^{NADH} = 0.006$ mM [45]

$k_m^{13P2G} = 0.01$ mM [44]

GK (Glucokinase)

$$Glc + ATP \rightarrow Glc6P + ADP$$

$$v_{GK} = V_{max}^{GK} \cdot \frac{ATP}{ATP + k_m^{ATP}} \cdot \frac{(Glc)^n}{(Glc)^n + (k_m^{Glc})^n}$$

$$V_{max}^{GK} = V_0^{GK} \cdot \frac{(Glc)^{n2}}{(Glc)^{n2} + (k_a^{Glc})^{n2}} \cdot \left(1 - f \cdot \frac{Fru6P}{Fru6P + k_i^{Fru6P}}\right)$$

$V_0^{GK} = 1.05 \cdot 10^4 mM\ h^{-1}$

$n = 1.5$ [46]

$K_m^{Glc} = 9\ mM$ [46]

$k_m^{ATP} = 0.55\ mM$ [47]

$k_i^{Fru6P} = 0.005\ mM$ [48]

$f = 0.75$ [48]

$n2 = 3.7$ [49]

$k_a^{Glc} = 15.9\ mM$ [49]

GlcT_{ER} (Glucose transport to ER)

$Glc_{ER} \leftrightarrow Glc$

$$v_{GlcT_{ER}} = V_{max}^{GlcT_{ER}} * \frac{(Glc - Glc_{ER})}{1 + \frac{Glc}{k_m^{Glc}} + \frac{Glc_{ER}}{k_m^{Glc_{ER}}}}$$

$V_{max}^{Glc_{ER}} = 1.94 \cdot 10^{10}\ h^{-1}$

$k_m^{Glc_{ER}} = 1.37\ mM$ [50, 51]

$k_m^{Glc} = 1.22\ mM$ [50, 51]

GLUT2 (Glucose transporter 2)

$Glc_{ext} \leftrightarrow Glc$

$$v_{GLUT2} = V_{max}^{GLUT2} \cdot \frac{Glc_{ext} - Glc}{1 + \frac{Glc_{ext}}{k_m^{Glc_{ext}}} + \frac{Glc}{k_m^{Glc}}}$$

$k_m^{Glc} = 17.3\ mM$ [52]

$k_m^{Glc_{ext}} = 17.3\ mM$ [52]

$V_{max}^{GLUT2} = 9.09 \cdot 10^1\ h^{-1}$

GP (Glycogen phosphorylase) [53, 54]

$$Glyc + P \leftrightarrow Glc1P$$

$$v_{GP} = V_{max}^{GP} \cdot \left((1-\gamma^{GP}) \cdot v_{GP}^{native} + \gamma^{GP} \cdot v_{GP}^{phospho}\right)$$

$$V_{max}^{GP} = 1.29 \cdot 10^{2} \cdot \left(\cdot \frac{Glyc}{store}\right) \; mM \cdot h^{-1}$$

$$store = 300 \; mM$$

$$v_{GP}^{native} = V_{max_{native}}^{GP} \cdot \frac{Glyc \cdot P - Glc1P/k_{eq}^{GP}}{\left(1+\frac{Glyc}{k_{m_{native}}^{Glyc}}\right)\cdot\left(1+\frac{P}{k_{m_{native}}^{P}}\right)+\left(1+\frac{Glc1P}{k_{m_{native}}^{Glc1P}}\right)-1}$$

$$V_{max_{native}}^{GP} = V_{0_{native}} \cdot \left(\frac{AMP}{AMP + K_{a_{native}}^{AMP}}\right)$$

$$V_{0_{native}} = \frac{1}{k_{m_{native}}^{Glyc} \cdot k_{m_{native}}^{P}}$$

$K_{a_{native}}^{AMP}$ = 0.36 mM [53]

$k_{eq}^{GP} = 0.21(mM)^{-1}$ [55]

$k_{m_{native}}^{Glyc}$ = 2.5 mM [54]

$k_{m_{native}}^{P} = 500 \; mM$ [54]

$$K_{m_{native}}^{Glc1P} = K_{0}^{Glc1P} \cdot \left(1 - \frac{AMP}{AMP + K_{a_{Glc1P}}^{AMP}}\right)$$

K_{0}^{Glc1P} = 250 mM [54]

$K_{a_{Glc1P}}^{AMP} = 0.5 \; mM$ [54]

$$v_{GP}^{phospho} = V_{max_{phospho}}^{GP} \cdot \frac{Glyc \cdot P - Glc1P/k_{eq}^{GP}}{\left(1+\frac{Glyc}{k_{m_{phospho}}^{Glyc}}\right)\cdot\left(1+\frac{P}{k_{m_{phospho}}^{P}}\right)+\left(1+\frac{Glc1P}{k_{m_{phospho}}^{Glc1P}}\right)-1}$$

$$V_{max^{phospho}}^{GP} = V_{0_{phospho}} \cdot \left(\frac{AMP}{AMP + K_{a_{phospho}}^{AMP}}\right)$$

$$V_{0_{phospho}} = \frac{1}{k_{m_{phospho}}^{Glyc} \cdot k_{m_{phospho}}^{P}}$$

$K_{a_{phospho}}^{AMP}$ = 0.017 mM [53]

$k_{m_{phospho}}^{Glyc} = 1.8\, mM$ [54]

$k_{m_{phospho}}^{P}$ = 2.1 mM [54]

$k_{m_{phospho}}^{Glc1P} = 0.7\, mM$ [54]

$G6P_{ER}$ (Glucose-6-phosphate phosphatase in the ER) [56, 57]

$$Glc6P_{ER} \rightarrow Glc_{ER} + P$$

$$v_{G6P\,ER} = V_{max}^{G6P_{ER}} \cdot \frac{Glc6P_{ER}}{Glc6P_{ER} + k_m^{Glc6P_{ER}}}$$

$k_m^{Glc6P_{ER}}$ = 1.84 mM [56]

$V_{max}^{G6P_{ER}} = 4.57 \cdot 10^2\, mM \cdot h^{-1}$

GPI (Glucose-6-phosphate isomerase)

$$Glc6P \leftrightarrow Fru6P$$

$$v_{GPI} = V_{max}^{GPI} \cdot \frac{Glc6P - Fru6P/k_{eq}^{GPI}}{1 + \frac{Glc6P}{k_m^{Glc6P}} + \frac{Fru6P}{k_m^{Fru6P}}}$$

$V_{max}^{GPI} = 1.07 \cdot 10^9 h^{-1}$

$k_{eq}^{GPI} =$ 0.3 [58]

$k_m^{Glc6P} = 0.055\, mM$ [59]

$K_m^{Fru6P_{cyt}}$ = 0.12 mM [59]

G1PI (Glucose-1-phosphate isomerase) [60]

$$Glc1P \leftrightarrow Glc6P$$

$$v_{G1PI} = v_{max}^{G1PI} \cdot \frac{Glc1P - Glc6P/k_{eq}^{G1PI}}{1 + \frac{Glc1P}{k_m^{Glc1P}} + \frac{Glc6P}{k_m^{Glc6P}}}$$

$v_{max}^{G1PI} = 7.78 \cdot 10^7 h^{-1}$

k_{eq}^{G1PI} =16.2 [61]

$k_m^{Glc1P} = 0.045$ mM [60]

$k_m^{Glc6P} = 0.67 mM$ [60]

G6PT$_{ER}$ (Glucose-6-phosphate transport to ER)

$$Glc6P_{ER} \leftrightarrow Glc6P$$

$$v_{G6PT_{ER}} = V_{max}^{G6PT_{ER}} \cdot \frac{(Glc6P - Glc6P_{ER})}{1 + \frac{Glc6P}{k_m^{Glc6P}} + \frac{Glc6P_{ER}}{k_m^{Glc6P_{ER}}}}$$

$V_{max}^{G6PT_{ER}} = 1.94 \cdot 10^{10}\ h^{-1}$

$k_m^{Glc6P_{ER}} = 1.12\ mM$ [50]

$k_m^{Glc6P} = 1.12\ mM$ [50]

GS (Glycogen synthase)

$$UDP\text{-}Glc \rightarrow UDP + Glyc$$

$$v_{GS} = V_{max}^{GS} \cdot \left((1 - \gamma^{GS}) \cdot v_{GS}^{native} + \gamma^{GS} \cdot v_{GS}^{phospho}\right)$$

$$V_{max}^{GS} = 1.16 \cdot 10^2 \frac{(store - glyc)}{(store - glyc) + 0.1 \cdot store} mM \cdot h^{-1}$$

$store = \ 300\ mM\ (average\ hepatocyte)$

$$v_{GS}^{native} = \frac{UDP\text{-}Glc}{UDP\text{-}Glc + \ K_{m-native}^{UDP\text{-}Glc}}$$

$$K_{m_{native}}^{UDP\text{-}Glc} = K_{0_{native}}^{UDP\text{-}Glc} \cdot \left(1 - \frac{Glc6P}{Glc6P + K_{a_{native}}^{Glc6p}}\right) + K_{b_{native}}^{UDP\text{-}Glc}$$

$K_{0_{native}}^{UDP\text{-}Glc} = 1.4$ mM [62]

$K_{a_{native}}^{Glc6P} = 0.007$ mM [62]

$K_{b_{native}}^{UDP\text{-}Glc} = 0.2$ mM [62]

$$v_{GS}^{phospho} = \frac{UDP\text{-}Glc}{UDP\text{-}Glc + \ K_{m_{phospho}}^{UDP\text{-}Glc}}$$

$$k_{m_{phospho}}^{UDP\text{-}Glc} = K_{0_{phospho}}^{UDP\text{-}Glc} \cdot \left(1 - \frac{Glc6P}{Glc6P + K_{a_{phospho}}^{Glc6P}}\right) + K_{b_{phospho}}^{UDP\text{-}Glc}$$

$K_{0_{phospho}}^{UDP\text{-}Glc} = 32$ mM [62]

$K_{a_{phospho}}^{Glc6P} = 0.09$ mM [62]

$K_{b_{phospho}}^{UDP\text{-}Glc} = 0.3$ mM [62]

LacT (Lactate transporter)

$Lac_{ext} \leftrightarrow Lac$

$$v_{LacT} = v_{max}^{LacT} \cdot \frac{Lac_{ext} - Lac}{1 + \frac{Lac}{k_m^{Lac}} + \frac{Lac_{ext}}{k_m^{Lac_{ext}}}}$$

$v_{max}^{LacT} = 5.83 \cdot 10^2\ h^{-1}$

$k_m^{Lac} = 2.42\ mM$ [63]

$k_m^{Lac_{ext}} = 2.42\ mM$ [63]

LDH (Lactate dehydrogenase) [64, 65]

$Pyr + NADH \leftrightarrow Lac + NAD^+$

$$v_{LDH} = v_{max}^{LDH} * \frac{Pyr \cdot NADH - Lac \cdot NAD^+ / k_{eq}^{LDH}}{\left(1 + \frac{NADH}{k_m^{NADH}}\right) \cdot \left(1 + \frac{Pyr}{k_m^{Pyr}}\right) + \left(1 + \frac{Lac}{k_m^{Lac}}\right) \cdot \left(1 + \frac{NAD^+}{k_m^{NAD^+}}\right) - 1}$$

$v_{max}^{LDH} = 1.56 \cdot 10^{11} h^{-1} \cdot mM^{-1}$

$k_{eq}^{LDH} = 9000$ [66]

$k_m^{NADH} = 0.015$ mM [65]

$k_m^{Pyr} = 0.15\ mM$ [65]

$k_m^{Lac} = 36\ mM$ [64]

$k_m^{NAD^+} = 0.11$ mM [65]

MalT (Malate transporter)

$$Mal_{mito} + P \leftrightarrow Mal + P_{mito}$$

$$v_{MalT} = v_{max}^{MalT} \cdot \left(\frac{Mal_{mito} \cdot P - Mal \cdot P_{mito}}{\left(1 + \frac{Mal_{mito}}{K_m^{Mal_{mito}}}\right) \cdot \left(1 + \frac{P}{K_m^P}\right) + \left(1 + \frac{Mal}{K_m^{Mal}}\right) \cdot \left(1 + \frac{P_{mito}}{K_m^{P_{mito}}}\right) - 1} \right)$$

$v_{max}^{MalT} = 1.94 \cdot 10^3 h^{-1} \cdot mM^{-1}$

k_m^P = 1.41 mM [67]

$k_m^{Mal_{mito}}$ = 0.49 mM [67]

$k_m^{P_{mito}}$ = 1.41 mM [67]

k_m^{Mal} = 0.49 mM [67]

MDH (Malate dehydrogenase)

$$Mal + NAD^+ \leftrightarrow OA + NADH$$

$$v_{MDH} = v_{max}^{MDH} \cdot \frac{Mal \cdot NAD^+ - OA \cdot NADH / k_{eq}^{MDH}}{\left(1 + \frac{Mal}{k_m^{Mal}}\right) \cdot \left(1 + \frac{NAD^+}{k_m^{NAD^+}}\right) + \left(1 + \frac{OA}{k_m^{OA}}\right) \cdot \left(1 + \frac{NADH}{k_m^{NADH}}\right) - 1}$$

$v_{max}^{MDH} = 1.94 \cdot 10^9 h^{-1} \cdot mM^{-1}$

$k_{eq}^{MDH} = 3 \cdot 10^{-5}$ [68]

$k_m^{Mal} = 1.1\, mM$ [69]

$k_m^{NAD^+} = 0.114\, mM$ [69]

$k_m^{OA} = 0.088\, mM$ [69]

$k_m^{NADH} = 0.026\, mM$ [69]

MDH$_{mito}$ (Mitochondrial malate dehydrogenase)

$$Mal_{mito} + NAD_{mito}^+ \leftrightarrow OA_{mito} + NADH_{mito}$$

$$v_{MDH_{mito}} = V_{max}^{MDH_{mito}}$$

$$\cdot \left(\frac{Mal_{mito} \cdot NAD_{mito}^{+} - {}^{1}\!\big/_{K_{eq}^{MDH_{mito}}} \cdot OA_{mito} \cdot NADH_{mito}}{\left(1+\frac{Mal_{mito}}{K_m^{Mal_{mito}}}\right)\cdot\left(1+\frac{NAD_{mito}}{K_m^{NAD_{mito}}}\right)+\left(1+\frac{OA_{mito}}{K_m^{OA_{mito}}}\right)\cdot\left(1+\frac{NADH_{mito}}{K_m^{NADH_{mito}}}\right)-1} \right)$$

$V_{max}^{MDH_{mito}} = 6.80 \cdot 10^{11} h^{-1} \cdot mM^{-1}$

$K_{eq}^{MDH_{mito}} = 3.1 \cdot 10^{-5}$ (pH 7.5) [68]

$K_m^{Mal_{mito}} = 0.33$ mM [70]

$K_m^{NAD_{mito}} = 0.06$ mM [71]

$K_m^{OA_{mito}} = 0.017$ mM [71]

$K_m^{NADH_{mito}} =$0.044 mM [71]

NDK^{GTP}, NDK^{UTP}, NDK$^{GTP}_{mito}$ (Cytosolic and mitochondrial nucleoside-diphosphate kinases)

$ATP_{mito} + GDP_{mito} \leftrightarrow ADP_{mito} + GTP_{mito}$

$ATP + GDP \leftrightarrow ADP + GTP$

$ATP + UDP \leftrightarrow ADP + UTP$

$$v_{NDK^{GTP}} = v_{max}^{NDK^{GTP}} \cdot \frac{ATP \cdot GDP - ADP \cdot GTP/k_{eq}^{NDK}}{\left(1+\frac{ATP}{k_m^{ATP}}\right)\left(1+\frac{GDP}{k_m^{GDP}}\right)+\left(1+\frac{ADP}{k_m^{ADP}}\right)\left(1+\frac{GTP}{k_m^{GTP}}\right)-1}$$

$$v_{NDK^{UTP}} = v_{max}^{NDK^{UTP}} \cdot \frac{ATP \cdot UDP - ADP \cdot UTP/k_{eq}^{NDK}}{\left(1+\frac{ATP}{k_m^{ATP}}\right)\left(1+\frac{UDP}{k_m^{UDP}}\right)+\left(1+\frac{ADP}{k_m^{ADP}}\right)\left(1+\frac{UTP}{k_m^{UTP}}\right)-1}$$

$$v_{NDK^{GTP}{}_{mito}} = v_{max}^{Ndk^{GTP}{}_{mito}} \cdot \frac{ATP_{mito} \cdot GDP_{mito} - ADP_{mito} \cdot GTP_{mito}/k_{eq}^{NDK}}{\left(1+\frac{ATP_{mito}}{k_m^{ATP_{mito}}}\right)\left(1+\frac{GDP_{mito}}{k_m^{GDP_{mito}}}\right)+\left(1+\frac{ADP_{mito}}{k_m^{ADP_{mito}}}\right)\left(1+\frac{GTP_{mito}}{k_m^{GTP_{mito}}}\right)-1}$$

$v_{max}^{NDK^{GTP}} = 1.94 \cdot 10^{11} h^{-1} \cdot mM^{-1}$

$v_{max}^{NDK^{UTP}} = 1.94 \cdot 10^{7} h^{-1} \cdot mM^{-1}$

$v_{max}^{NDK^{GTP}mito} = 1.94 \cdot 10^{7} h^{-1} \cdot mM^{-1}$

$k_{eq}^{NDK} = 1$ [72]

$k_m^{ATP} = 1.33\ mM$ [73]

$k_m^{GDP} = 3.1 * 10^{-2}\ mM$[73]

$k_m^{ADP} = 4.2 * 10^{-2}\ mM$ [73]

$k_m^{GTP} = 0.15\ mM$ [74]

$K_m^{ATP_{mito}} = 1.66$ mM [73]

$K_m^{GDP_{mito}} = 0.036$ mM [73]

$K_m^{ADP_{mito}} = 0.073$ mM [73]

$K_m^{GTP\_mito} = 0.15$ mM [74]

$k_m^{UTP} = 16\ mM$ [74]

$k_m^{UDP} = 0.19\ mM$ [73]

PC (Pyruvate carboxylase) [75, 76]

$$ATP_{mito} + Pyr_{mito} + CO_{2_{mito}} \leftrightarrow OA_{mito} + ADP_{mito} + P_{mito}$$

$$v_{PC} = v_{max}^{PC} \cdot \frac{ATP_{mito} \cdot Pyr_{mito} \cdot CO_{2_{mito}} - OA_{mito} \cdot ADP_{mito} \cdot P_{mito}/k_{eq}^{PC}}{\left(ATP_{mito} + k_m^{ATP_{mito}}\right) \cdot \left(Pyr_{mito} + k_m^{Pyr_{mito}}\right) \cdot \left(CO_{2_{mito}} + k_m^{CO_{2_{mito}}}\right)}$$

$v_{max}^{PC} = 3.59 \cdot 10^3 mM \cdot h^{-1}$

$k_m^{ATP_{mito}} = 0.14$ mM [75]

$k_m^{Pyr_{mito}} = 0.33$ mM [75]

$k_m^{CO_{2_{mito}}} = 4.2$ mM [75]

$k_{eq}^{PC} = 6.55$ [76]

PEPCK (Phosphoenolpyruvate carboxykinase)

$$OA + GTP \leftrightarrow PEP + GDP + CO_2$$

$$v_{PEPCK} = v_{max}^{PEPCK} \cdot \frac{OA \cdot GTP - PEP \cdot GDP * CO_2/k_{eq}^{PEPCK}}{\left(1+\frac{OA}{k_m^{OA}}\right)\cdot\left(1+\frac{GTP}{k_m^{GTP}}\right)+\left(1+\frac{PEP}{k_m^{PEP}}\right)\cdot\left(1+\frac{GDP}{k_m^{GDP}}\right)\cdot\left(1+\frac{CO_2}{k_m^{CO_2}}\right)-1}$$

$v_{max}^{PEPCK} = 5.11 \cdot 10^5 h^{-1} \cdot mM^{-1}$

k_{eq}^{PEPCK} =110 mM [77]

$k_m^{OA} =$ 0.024 mM [78]

k_m^{GTP} =0.021 mM [79]

$k_m^{PEP} = 0.4\ mM$ [80]

k_m^{GDP} = 0.02 mM [81]

$k_m^{CO_2}$ = 1.194 mM [82]

PEPCK$_{mito}$ (Mitochondrial phosphoenolpyruvate carboxykinase)

$$OA_{mito} + GTP_{mito} \leftrightarrow PEP_{mito} + GDP_{mito} + CO_{2_{mito}}$$

$$v_{PEPCK_{mito}} = v_{max}^{PEPCK_{mito}} \cdot \frac{OA_{mito} \cdot GTP_{mito} - PEP_{mito} \cdot GDP_{mito} \cdot CO_{2_{mito}}/k_{eq}^{PEPCK_{mito}}}{\left(1+\frac{OA_{mito}}{k_{m^{mito}}^{OA}}\right)\cdot\left(1+\frac{GTP_{mito}}{k_{m^{mito}}^{GTP}}\right)+\left(1+\frac{PEP_{mito}}{k_m^{PEP_{mito}}}\right)\cdot\left(1+\frac{GDP_{mito}}{k_m^{GDP_{mito}}}\right)\cdot\left(1+\frac{CO_{2_{mito}}}{k_m^{CO_{2_{mito}}}}\right)-1}$$

$v_{max}^{PEPCK_{mito}} = 1.0 \cdot 10^6 h^{-1} \cdot mM^{-1}$

$k_{eq}^{PEPCK_{mito}}$ =160 mM [77]

$k_m^{OA_{mito}}$ = 0.0085 mM [83]

$k_m^{GTP_{mito}}$ = 0.022 mM [81]

$k_m^{PEP_{mito}} = 0.4\ mM$ [80]

$k_m^{GDP_{mito}}$ = 0.02 mM [81]

$k_m^{CO_{2_{mito}}}$ = 1.06 mM [84]

PEPT (Phosphoenolpyruvate transporter)

$$PEP \leftrightarrow PEP_{mito}$$

$$v_{PEPT} = v_{max}^{PEPT} \cdot \frac{PEP_{mito} - PEP/k_{eq}^{PEPT}}{1 + \frac{PEP}{k_m^{PEP}} + \frac{PEP_{mito}}{k_m^{PEP_{mito}}}}$$

$v_{max}^{PEPT} = 1.94 \cdot 10^5\ h^{-1}$

$k_{eq}^{PEPT} = \exp\left(-\frac{Vmm \cdot F}{R \cdot T}\right)$

$k_m^{PEP} = 0.1\ mM$ [85]

$k_m^{PEP_{mito}} = 0.1\ mM$ [85]

PFK1 (Phosphofructokinase 1)

$$Fru6P + ATP \rightarrow Fru16P + ADP$$

$$v_{PFK1} = v_{max}^{PFK1} \cdot \frac{ATP}{ATP + K_m^{ATP}} \cdot \left(1 - \frac{ATP^{n_i}}{ATP^{n_i} + \left(K_i^{ATP}\right)^{n_i}}\right) \cdot \frac{(Fru6P)^{n_{Fru6P}}}{(Fru6P)^{n_{Fru6P}} + (k_m^{Fru6P})^{n_{Fru6P}}}$$

$v_{max}^{PFK1} = 7.68 \cdot 10^4 mM \cdot h^{-1}$

$K_m^{ATP} = K_0^{ATP} \cdot \left(1 - \frac{Fru26P_2}{Fru26P_2 + K_a^{Fru26P_2}}\right)$

$K_0^{ATP} = 0.2$ mM [86, 87]

$K_a^{Fru26P_2} = 0.0027$ mM [86, 87]

$K_i^{ATP} = K_{i0}^{ATP} \cdot \left(1 + f_{Fru26P_2} \frac{Fru26P_2}{Fru26P_2 + K_{a2}^{Fru26P_2}}\right)$

$K_{i0}^{ATP} = 0.7$ mM [86, 87]

$f_{Fru26P_2} = 9$ [86, 87]

$K_{a2}^{Fru26P_2} = 0.54\ mM$ [86, 87]

$n_i = 4$ [86, 87]

$$k_m^{Fru6P} = K_0^{Fru6p} \cdot \left(1 + \frac{ATP}{k_i^{ATP}}\right) \cdot \left(1 + \frac{Cit}{k_i^{Cit}}\right) \cdot \left(1 - f_{AMP} \frac{AMP^{n_{AMP}}}{AMP^{n_{AMP}} + (K_a^{AMP})^{n_{AMP}}}\right)$$

$$\cdot \left(1 - f_P \frac{P}{P + K_a^P}\right) \cdot \left(1 - f_{Fru26P_2} \frac{Fru26P_2^{n_{Fru26P_2}}}{Fru26P_2^{n_{Fru26P_2}} + \left(K_a^{Fru26P_2}\right)^{n_{Fru26P_2}}}\right)$$

$K_0^{Fru6P} = 1.14$ mM [88]

$k_i^{ATP} = 0.6$ mM [88]

$k_i^{Cit} = 3.27$ mM [88]

$f_{AMP} = 0.77$ [88]

$K_a^{AMP} = 0.1$ mM [88]

$n_{AMP} = 1.84$ [88]

$f_P = 0.85$ [88]

$K_a^P = 0.69$ mM [88]

$f_{Fru26P_2} = 0.92$ [86]

$K_a^{Fru26P_2} = 0.0045\, mM$ [86]

$n_{Fru26P_2} = 1.2$ [86]

$$n^{Fru6P} = \left(n_0 + \frac{ATP^{n_{ATP}}}{ATP^{n_{ATP}} + \left(K_i^{ATP}\right)^{n_{ATP}}}\right) \cdot \left(1 - f_{AMP} \frac{AMP^{n_{AMP}}}{AMP^{n_{AMP}} + (K_a^{AMP})^{n_{AMP}}}\right)$$

$$\cdot \left(1 + f_{Cit} \frac{Cit^{n_{Cit}}}{Cit^{n_{Cit}} + \left(K_i^{Cit}\right)^{n_{Cit}}}\right) \cdot \left(1 - f_P \frac{P^{n_P}}{P^{n_P} + (K_a^P)^{n_P}}\right) \cdot$$

$$\cdot \left(1 - f_{Fru26P_2} \frac{Fru26P_2^{\;n_{Fru26bp}}}{Fru26P_2^{n_{Fru26P_2}} + \left(K_a^{Fru26P_2}\right)^{n_{Fru26P_2}}}\right)$$

$n_0 = 3.67$ [88]

$K_i^{ATP} = 0.13$ mM [88]

$n_{ATP} = 1.59$ [88]

$f_{AMP} = 0.4$ [88]

$K_a^{AMP} = 0.086$ mM [88]

$n_{AMP} = 2.22$ [88]

$f_{Cit} = 0.1$ [88]

$K_i^{Cit} = 0.18$ mM [88]

$n_{Cit} = 4$ [88]

$f_P = 0.28$ [88]

$K_a^P = 0.53$ mM [88]

$n_P = 4$ [88]

$f_{Fru26P_2} = 0.37$ [86]

$K_a^{Fru26P_2} = 0.0021$ mM [86]

$n^{Fru26P_2} = 4$ [86]

PFK2/FBP2 (Phosphofructokinase 2/Fructose-2,6-bisphosphatase)

$$\boldsymbol{Fru6P + ATP \rightarrow Fru26P_2 + ADP}$$

$$v_{PFK2} = (1-\gamma^{PFK2}) \cdot v_{PFK2}^{native} + \gamma^{PFK2} \cdot v_{PFK2}^{phospho}$$

$$v_{PFK2}^{native} = V_{\max}^{PFK2} \frac{Fru6P^n}{Fru6P^n + \left(k_{m^{native}}^{Fru6P}\right)^n} \cdot \frac{ATP}{ATP + k_m^{ATP}} \cdot \left(1 - n_0 \cdot \frac{PEP}{PEP + k_i^{PEP}}\right)$$

$V_{max}^{PFK2} = 1.51 \cdot 10^2\ mM \cdot h^{-1}$

$k_{m^{native}}^{Fru6P} = 0.015$ mM [89]

$n = 1.3$ [89]

$k_m^{ATP} = 0.25\ mM$ [89]

$k_i^{PEP} = 0.25\ mM$ [90]

$n_0 = 0.85$ [90]

$$v_{PFK2}^{phospho} = V_{max}^{PFK2} \frac{Fru6P^n}{Fru6P^n + {k_{m^{phospho}}^{Fru6P}}^n} \cdot \frac{ATP}{ATP + k_m^{ATP}} \cdot \left(1 - n_0 \cdot \frac{PEP}{PEP + k_i^{PEP}}\right)$$

$k_{m^{phospho}}^{Fru6P} = 0.05$ mM [89]

$n = 2$ [89]

$k_m^{ATP} = 0.5\, mM$ [89]

$k_i^{PEP} = 0.25\, mM$ [90]

$n_0 = 0.85$ [90]

$\boldsymbol{Fru26P_2 \rightarrow Fru6P + P}$

$$v_{FBP2} = V_{max}^{FBP2} * \left((1-\gamma^{FBP2}) \cdot v_{FBP2}^{native} + \gamma^{FBP2} \cdot v_{FBP2}^{phospho}\right)$$

$$V_{max}^{FBP2} = 5.49 \cdot 10^2\, mM \cdot h^{-1}$$

$$v_{FBP2}^{native} = \frac{Fru26P_2}{Fru26P_2 + k_{m^{native}}^{Fru26P_2}} \Big/ \left(1 + \frac{Fru6P}{k_{i_{native}}^{Fru6P}}\right)$$

$k_{m^{native}}^{Fru26P_2} = 0.01\, mM$ [91]

$k_{i_{native}}^{Fru6P} = 0.0035$ mM [89]

$$v_{FBP2}^{phospho} = \frac{Fru26P_2}{Fru26P_2 + k_{m^{phospho}}^{Fru26P_2}} \Big/ \left(1 + \frac{Fru6P}{k_{i_{phospho}}^{Fru6P}}\right)$$

$k_{m^{phospho}}^{Fru26P_2} = 0.0005$ mM [89]

$k_{i_{phospho}}^{Fru6P} = 0.01\, mM$ [89]

PGK (Phosphoglycerate kinase)

$ADP + 13P2G \rightarrow ATP + 3PG$

$$v_{PGK} = v_{max}^{PGK} * \frac{ADP \cdot 13P2G - ATP \cdot 3PG / k_{eq}^{PGK}}{\left(1 + \frac{ADP}{k_m^{ADP}}\right) \cdot \left(1 + \frac{13P2G}{k_m^{13P2G}}\right) + \left(1 + \frac{ATP}{k_m^{ATP}}\right) \cdot \left(1 + \frac{3PG}{k_m^{3PG}}\right) - 1}$$

$v_{max}^{PGK} = 1.94 \cdot 10^{10} h^{-1} \cdot mM^{-1}$

$k_{eq}^{PGK} = 1830$ [92]

$k_m^{ADP} = 0.35\, mM$ [93]

$k_m^{13P2G} = 0.0022\, mM$ [93]

$k_m^{ATP} = 0.24\, mM$ [94]

$k_m^{3PG} = 1.65\ mM$ [94]

PGM (Phosphoglycerate mutase)

$3PG \leftrightarrow 2PG$

$$v_{PGM} = v_{max}^{PGM} \cdot \frac{3PG - 2PG/k_{eq}^{PGM}}{1 + \frac{3PG}{k_m^{3PG}} + \frac{2PG}{K_m^{2PG}}}$$

$v_{max}^{PGM} = 1.94 \cdot 10^{10}\ h^{-1}$

$k_{eq}^{PGM} = 0.096$ [95]

$k_m^{3PG} = 0.52\ mM$ [96]

$K_m^{2PG} = 0.24\ mM$ [96]

PK (Pyruvate kinase)

$PEP + ADP \leftrightarrow Pyr + ATP$

$$v_{PK} = v_{max}^{PK} \cdot \left((1 - \gamma^{PK}) \cdot v_{PK}^{native} + \gamma^{PK} \cdot v_{PK}^{phospho}\right)$$

$$v_{PK}^{native} = \frac{PEP}{PEP + k_{m^{native}}^{PEP} \cdot \left(1 + \frac{ATP}{k_{i_{native}}^{ATP}}\right) \cdot \left(1 - \frac{Fru16P_2}{Fru16P_2 + k_{a^{native}}^{Fru16P_2}}\right)} \cdot \frac{ADP}{ADP + k_m^{ADP}}$$

$v_{max}^{PK} = 1.28 \cdot 10^4\ mM \cdot h^{-1}$

$k_{m^{native}}^{PEP}$ =0.13 mM [97]

$k_{i_{native}}^{ATP}$ =1 mM [97]

$k_{a^{native}}^{Fru16P_2}$ =0.0078 mM [98]

k_m^{ADP} = 0.25 mM [99]

$$v_{PK}^{phospho} = \frac{PEP^n}{PEP^n + \left(k_{m^{phospho}}^{PEP} \cdot \left(1 + \frac{ATP}{k_{i_{phospho}}^{ATP}}\right) \cdot \left(1 - \frac{Fru16P_2}{Fru16P_2 + k_{a^{phospho}}^{Fru16P_2}}\right)\right)^n} \cdot \frac{ADP}{ADP + k_m^{ADP}}$$

$k_{m^{phospho}}^{PEP}$ = 5.8 mM [97]

$n = 2.9$ [97]

$k_{a_{phospho}}^{Fru16P_2} = 0.0095$ mM [98]

$k_{i_{phospho}}^{ATP} = 0.32\ mM$ [32]

$k_m^{ADP} = 0.33$ mM [99]

PyrMalT (Pyruvate/malate antiporter)

$Mal_{mito} + Pyr \leftrightarrow Mal + Pyr_{mito}$

$$v_{PyrMalT} = v_{max}^{PyrMalT} \cdot \left(\frac{Mal_{mito} \cdot Pyr - Mal \cdot Pyr_{mito}}{\left(1 + \frac{Mal_{mito}}{K_m^{Mal_{mito}}}\right) \cdot \left(1 + \frac{Pyr}{K_m^{Pyr}}\right) + \left(1 + \frac{Mal}{K_m^{Mal}}\right) \cdot \left(1 + \frac{Pyr_{mito}}{K_m^{Pyr_{mito}}}\right) - 1} \right)$$

$v_{max}^{PyrMalT} = 1.94 \cdot 10^4\ h^{-1} \cdot mM^{-1}$

$k_m^{Pyr} = 0.84\ mM$ [100]

$k_m^{Mal} = 0.7\ mM$ [85]

$k_m^{Pyr_{mito}} = 0.84\ mM$ [100]

$k_m^{Mal_{mito}} = 0.7\ mM$ [85]

PyrT (Pyruvate transporter)

$Pyr \leftrightarrow Pyr_{mito}$

$$v_{PyrT} = v_{max}^{PyrT} \cdot \frac{Pyr \cdot H^+ - Pyr_{mito} \cdot H_{mito}^+}{1 + \frac{Pyr}{k_m^{Pyr}} + \frac{Pyr_{mito}}{k_m^{Pyr_{mito}}}}$$

$v_{max}^{PyrT} = 1.94 \cdot 10^8 h^{-1} \cdot mM^{-1}$

$k_m^{Pyr} = 0.15$ mM [101]

$k_m^{Pyr_{mito}} = 0.15$ mM [101]

TPI (Triosephosphate isomerase)

$DHAP \leftrightarrow GAP$

$$v_{TPI} = v_{max}^{TPI} \cdot \frac{DHAP - GAP/k_{eq}^{TPI}}{1 + \frac{DHAP}{k_m^{DHAP}} + \frac{GAP}{k_m^{GAP}}}$$

$v_{max}^{TPI} = 1.94 \cdot 10^8\ h^{-1}$

k_{eq}^{TPI} = 0.04545 [37]

k_m^{DHAP} = 0.59 mM [102]

k_m^{GAP} = 0.415 mM [102]

UGT (Uridine diphospho-glucuronosyltransferase)

$UTP + Glc1P \leftrightarrow UDP\text{-}Glc + PP$

$$v_{UGT} = v_{max}^{UGT} * \frac{UTP * Glc1P - UDP\text{-}Glc * PP/k_{eq}^{UGT}}{\left(1 + \frac{UTP}{k_m^{UTP}}\right)\left(1 + \frac{Glc1P}{k_m^{Glc1P}}\right) + \left(1 + \frac{UDP\text{-}Glc}{k_m^{UDP\text{-}Glc}}\right)\left(1 + \frac{PP}{k_m^{PP}}\right) - 1}$$

$v_{max}^{UGT} = 7.78 \cdot 10^9 h^{-1} \cdot mM^{-1}$

k_{eq}^{UGT} = 0.3122 [103]

k_m^{UTP} = 0.2 mM [103]

k_m^{Glc1P} = 0.055 mM [103]

$k_m^{UDP\text{-}Glc}$ = 0.06 mM [103]

k_m^{PP} = 0.084 mM [103]

References

1. Tischler ME, Hecht P, Williamson JR: Determination of mitochondrial/cytosolic metabolite gradients in isolated rat liver cells by cell disruption. Archives of biochemistry and biophysics 1977, 181(1):278-293.
2. Siess EA, Brocks DG, Lattke HK, Wieland OH: Effect of glucagon on metabolite compartmentation in isolated rat liver cells during gluconeogenesis from lactate. Biochem J 1977, 166(2):225-235.
3. Albe KR, Butler MH, Wright BE: Cellular concentrations of enzymes and their substrates. Journal of theoretical biology 1990, 143(2):163-195.
4. Start C, Newsholme EA: The effects of starvation and alloxan-diabetes on the contents of citrate and other metabolic intermediates in rat liver. Biochem J 1968, 107(3):411-415.

5. Konig M, Bulik S, Holzhutter HG: Quantifying the contribution of the liver to glucose homeostasis: a detailed kinetic model of human hepatic glucose metabolism. PLoS Comput Biol 2012, 8(6):e1002577.
6. Jackson RC, Lui MS, Boritzki TJ, Morris HP, Weber G: Purine and pyrimidine nucleotide patterns of normal, differentiating, and regenerating liver and of hepatomas in rats. Cancer research 1980, 40(4):1286-1291.
7. Keppler DO, Pausch J, Decker K: Selective uridine triphosphate deficiency induced by D-galactosamine in liver and reversed by pyrimidine nucleotide precursors. Effect on ribonucleic acid synthesis. The Journal of biological chemistry 1974, 249(1):211-216.
8. Smith CM, Bryla J, Williamson JR: Regulation of mitochondrial alpha-ketoglutarate metabolism by product inhibition at alpha-ketoglutarate dehydrogenase. The Journal of biological chemistry 1974, 249(5):1497-1505.
9. Greenbaum AL, Gumaa KA, McLean P: The distribution of hepatic metabolites and the control of the pathways of carbohydrate metabolism in animals of different dietary and hormonal status. Archives of biochemistry and biophysics 1971, 143(2):617-663.
10. Lagunas R, McLean P, Greenbaum AL: The effect of raising the NAD+ content on the pathways of carbohydrate metabolism and lipogenesis in rat liver. Eur J Biochem 1970, 15(1):179-190.
11. Krebs HA: The redox state of nicotinamide adenine dinucleotide in the cytoplasm and mitochondria of rat liver. Advances in enzyme regulation 1967, 5:409-434.
12. Veech RL, Veloso D, Mehlman MA: Thiamin deficiency: liver metabolite levels and redox and phosphorylation states in thiamin-deficient rats. The Journal of nutrition 1973, 103(2):267-272.
13. Birt LM, Bartley W: The behaviour of pyridine nucleotides of mitochondria in a 'saline medium'. Biochem J 1960, 76:328-341.
14. Wilson DF, Stubbs M, Oshino N, Erecinska M: Thermodynamic relationships between the mitochondrial oxidation-reduction reactions and cellular ATP levels in ascites tumor cells and perfused rat liver. Biochemistry 1974, 13(26):5305-5311.
15. Aw TY, Andersson BS, Jones DP: Mitochondrial transmembrane ion distribution during anoxia. The American journal of physiology 1987, 252(4 Pt 1):C356-361.
16. Werkheiser WC, Bartley W: The study of steady-state concentrations of internal solutes of mitochondria by rapid centrifugal transfer to a fixation medium. Biochem J 1957, 66(1):79-91.
17. Guynn RW, Veloso D, Lawson JW, Veech RL: The concentration and control of cytoplasmic free inorganic pyrophosphate in rat liver in vivo. Biochem J 1974, 140(3):369-375.
18. Hitchings GH: Indications for control mechanisms in purine and pyrimidine biosynthesis as revealed by studies with inhibitors. Advances in enzyme regulation 1974, 12:121-129.
19. Keppler D, Frohlich J, Reutter W, Wieland O, Decker K: Changes in uridine nucleotides during liver perfusion with D-galactosamine. FEBS letters 1969, 4(4):278-280.
20. Mitchell P, Moyle J: Estimation of membrane potential and pH difference across the cristae membrane of rat liver mitochondria. Eur J Biochem 1969, 7(4):471-484.
21. la Fleur SE, Kalsbeek A, Wortel J, Fekkes ML, Buijs RM: A daily rhythm in glucose tolerance: a role for the suprachiasmatic nucleus. Diabetes 2001, 50(6):1237-1243.
22. Frangioudakis G, Gyte AC, Loxham SJ, Poucher SM: The intravenous glucose tolerance test in cannulated Wistar rats: a robust method for the in vivo assessment of glucose-stimulated insulin secretion. Journal of pharmacological and toxicological methods 2008, 57(2):106-113.
23. Hara E, Saito M: Diurnal changes in plasma glucose and insulin responses to oral glucose load in rats. The American journal of physiology 1980, 238(5):E463-466.

24. Balks HJ, Jungermann K: Regulation of peripheral insulin/glucagon levels by rat liver. Eur J Biochem 1984, 141(3):645-650.
25. Diaz B, Blazquez E: Effect of pinealectomy on plasma glucose, insulin and glucagon levels in the rat. Hormone and metabolic research = Hormon- und Stoffwechselforschung = Hormones et metabolisme 1986, 18(4):225-229.
26. Patel DG: Lack of glucagon response to hypoglycemia in long-term experimental diabetic rats. Diabetes 1983, 32(1):55-60.
27. Wan CK, Giacca A, Matsuhisa M, El-Bahrani B, Lam L, Rodgers C, Shi ZQ: Increased responses of glucagon and glucose production to hypoglycemia with intraperitoneal versus subcutaneous insulin treatment. Metabolism 2000, 49(8):984-989.
28. Zhou H, Tran PO, Yang S, Zhang T, LeRoy E, Oseid E, Robertson RP: Regulation of alpha-cell function by the beta-cell during hypoglycemia in Wistar rats: the "switch-off" hypothesis. Diabetes 2004, 53(6):1482-1487.
29. Bartrons R, Hue L, Van Schaftingen E, Hers HG: Hormonal control of fructose 2,6-bisphosphate concentration in isolated rat hepatocytes. Biochem J 1983, 214(3):829-837.
30. El-Maghrabi MR, Claus TH, Pilkis J, Fox E, Pilkis SJ: Regulation of rat liver fructose 2,6-bisphosphatase. The Journal of biological chemistry 1982, 257(13):7603-7607.
31. Pilkis S, Schlumpf J, Pilkis J, Claus TH: Regulation of phosphofructokinase activity by glucagon in isolated rat hepatocytes. Biochem Biophys Res Commun 1979, 88(3):960-967.
32. Feliu JE, Hue L, Hers HG: Hormonal control of pyruvate kinase activity and of gluconeogenesis in isolated hepatocytes. Proc Natl Acad Sci U S A 1976, 73(8):2762-2766.
33. Claus TH, El-Maghrabi MR, Pilkis SJ: Modulation of the phosphorylation state of rat liver pyruvate kinase by allosteric effectors and insulin. The Journal of biological chemistry 1979, 254(16):7855-7864.
34. Syed NA, Khandelwal RL: Reciprocal regulation of glycogen phosphorylase and glycogen synthase by insulin involving phosphatidylinositol-3 kinase and protein phosphatase-1 in HepG2 cells. Molecular and cellular biochemistry 2000, 211(1-2):123-136.
35. Hartmann H, Probst I, Jungermann K, Creutzfeldt W: Inhibition of glycogenolysis and glycogen phosphorylase by insulin and proinsulin in rat hepatocyte cultures. Diabetes 1987, 36(5):551-555.
36. Schudt C: Regulation of glycogen synthesis in rat-hepatocyte cultures by glucose, insulin and glucocorticoids. Eur J Biochem 1979, 97(1):155-160.
37. Veech RL, Raijman L, Dalziel K, Krebs HA: Disequilibrium in the triose phosphate isomerase system in rat liver. Biochem J 1969, 115(4):837-842.
38. Ikehara Y, Endo H, Okada Y: The identity of the aldolases isolated from rat muscle and primary hepatoma. Archives of biochemistry and biophysics 1970, 136(2):491-497.
39. Malay AD, Procious SL, Tolan DR: The temperature dependence of activity and structure for the most prevalent mutant aldolase B associated with hereditary fructose intolerance. Archives of biochemistry and biophysics 2002, 408(2):295-304.
40. Schuster R, Holzhutter HG: Use of mathematical models for predicting the metabolic effect of large-scale enzyme activity alterations. Application to enzyme deficiencies of red blood cells. Eur J Biochem 1995, 229(2):403-418.
41. Rider CC, Taylor CB: Enolase isoenzymes in rat tissues. Electrophoretic, chromatographic, immunological and kinetic properties. Biochimica et biophysica acta 1974, 365(1):285-300.

42. Meek DW, Nimmo HG: Effects of phosphorylation on the kinetic properties of rat liver fructose-1,6-bisphosphatase. Biochem J 1984, 222(1):125-130.
43. Cori CF, Velick SF, Cori GT: The combination of diphosphopyridine nucleotide with glyceraldehyde phosphate dehydrogenase. Biochimica et biophysica acta 1950, 4(1-3):160-169.
44. Ryzlak MT, Pietruszko R: Heterogeneity of Glyceraldehyde-3-Phosphate Dehydrogenase from Human-Brain. Biochimica et biophysica acta 1988, 954(3):309-324.
45. Smith CM, Velick SF: The glyceraldehyde 3-phosphate dehydrogenases of liver and muscle. Cooperative interactions and conditions for functional reversibility. The Journal of biological chemistry 1972, 247(1):273-284.
46. Bontemps F, Hue L, Hers HG: Phosphorylation of glucose in isolated rat hepatocytes. Sigmoidal kinetics explained by the activity of glucokinase alone. Biochem J 1978, 174(2):603-611.
47. Storer AC, Cornishbowden A: Kinetics of Rat-Liver Glucokinase - Cooperative Interactions with Glucose at Physiologically Significant Concentrations. Biochemical Journal 1976, 159(1):7-14.
48. Van Schaftingen E: A protein from rat liver confers to glucokinase the property of being antagonistically regulated by fructose 6-phosphate and fructose 1-phosphate. Eur J Biochem 1989, 179(1):179-184.
49. Agius L, Peak M: Intracellular binding of glucokinase in hepatocytes and translocation by glucose, fructose and insulin. Biochem J 1993, 296 (Pt 3):785-796.
50. Igarashi Y, Kato S, Tada K: Kinetic properties of the glucose-6-phosphate transport system in rat hepatic microsomal membranes. J Inherit Metab Dis 1985, 8(3):153-154.
51. St-Denis JF, Berteloot A, Vidal H, Annabi B, van de Werve G: Glucose transport and glucose 6-phosphate hydrolysis in intact rat liver microsomes. The Journal of biological chemistry 1995, 270(36):21092-21097.
52. Ciaraldi TP, Horuk R, Matthaei S: Biochemical and Functional-Characterization of the Rat-Liver Glucose-Transport System - Comparisons with the Adipocyte Glucose-Transport System. Biochemical Journal 1986, 240(1):115-123.
53. Stalmans W, Gevers G: The catalytic activity of phosphorylase b in the liver. With a note on the assay in the glycogenolytic direction. Biochem J 1981, 200(2):327-336.
54. Tan AW, Nuttall FQ: Characteristics of the dephosphorylated form of phosphorylase purified from rat liver and measurement of its activity in crude liver preparations. Biochimica et biophysica acta 1975, 410(1):45-60.
55. Maddaiah VT, Madsen NB: Kinetics of purified liver phosphorylase. The Journal of biological chemistry 1966, 241(17):3873-3881.
56. Arion WJ, Nordlie RC: Liver Microsomal Glucose 6-Phosphatase, Inorganic Pyrophosphatase, and Pyrophosphate-Glucose Phosphotransferase. Ii. Kinetic Studies. The Journal of biological chemistry 1964, 239:2752-2757.
57. Kelmer-Bracht AM, Santos CP, Ishii-Iwamoto EL, Broetto-Biazon AC, Bracht A: Kinetic properties of the glucose 6-phosphatase of the liver from arthritic rats. Biochimica et biophysica acta 2003, 1638(1):50-56.
58. Tewari YB, Steckler DK, Goldberg RN: Thermodynamics of isomerization reactions involving sugar phosphates. The Journal of biological chemistry 1988, 263(8):3664-3669.
59. Zalitis J, Oliver IT: Inhibition of Glucose Phosphate Isomerase by Metabolic Intermediates of Fructose. Biochemical Journal 1967, 102(3):753-&.

60. Kashiwaya Y, Sato K, Tsuchiya N, Thomas S, Fell DA, Veech RL, Passonneau JV: Control of Glucose-Utilization in Working Perfused Rat-Heart. Journal of Biological Chemistry 1994, 269(41):25502-25514.
61. Colowick SP, Sutherland EW: Polysaccharide synthesis from glucose by means purified enzymes. Journal of Biological Chemistry 1942, 144(2):423-437.
62. Westphal SA, Nuttall FQ: Comparative characterization of human and rat liver glycogen synthase. Archives of biochemistry and biophysics 1992, 292(2):479-486.
63. Edlund GL, Halestrap AP: The kinetics of transport of lactate and pyruvate into rat hepatocytes. Evidence for the presence of a specific carrier similar to that in erythrocytes. Biochem J 1988, 249(1):117-126.
64. Anderson SR, Florini JR, Vestling CS: Rat Liver Lactate Dehydrogenase. 3. Kinetics and Specificity. The Journal of biological chemistry 1964, 239:2991-2997.
65. Prabhakaram M, Singh SN: Effect of age on the crystalline rat liver lactate dehydrogenase. Arch Gerontol Geriatr 1986, 5(1):57-64.
66. Williamson DH, Lund P, Krebs HA: The redox state of free nicotinamide-adenine dinucleotide in the cytoplasm and mitochondria of rat liver. Biochem J 1967, 103(2):514-527.
67. Indiveri C, Capobianco L, Kramer R, Palmieri F: Kinetics of the Reconstituted Dicarboxylate Carrier from Rat-Liver Mitochondria. Biochimica et biophysica acta 1989, 977(2):187-193.
68. Raval DN, Wolfe RG: Malic Dehydrogenase .4. Ph Dependence of Kinetic Parameters. Biochemistry 1962, 1(6):1118-&.
69. Crow KE, Braggins TJ, Batt RD, Hardman MJ: Rat liver cytosolic malate dehydrogenase: purification, kinetic properties, role in control of free cytosolic NADH concentration. Analysis of control of ethanol metabolism using computer simulation. The Journal of biological chemistry 1982, 257(23):14217-14225.
70. Thorne CJR: Properties of Mitochondrial Malate Dehydrogenases. Biochimica et biophysica acta 1962, 59(3):624-&.
71. Gelpi JL, Dordal A, Montserrat J, Mazo A, Cortes A: Kinetic-Studies of the Regulation of Mitochondrial Malate-Dehydrogenase by Citrate. Biochemical Journal 1992, 283:289-297.
72. Lynn R, Guynn RW: Equilibrium constants under physiological conditions for the reactions of succinyl coenzyme A synthetase and the hydrolysis of succinyl coenzyme A to coenzyme A and succinate. The Journal of biological chemistry 1978, 253(8):2546-2553.
73. Kimura N, Shimada N: Membrane-Associated Nucleoside Diphosphate Kinase from Rat-Liver - Purification, Characterization, and Comparison with Cytosolic Enzyme. Journal of Biological Chemistry 1988, 263(10):4647-4653.
74. Fukuchi T, Shimada N, Hanai N, Ishikawa N, Watanabe K, Kimura N: Recombinant rat nucleoside diphosphate kinase isoforms (alpha and beta): purification, properties and application to immunological detection of native isoforms in rat tissues. Biochimica et biophysica acta 1994, 1205(1):113-122.
75. Wimhurst JM, Manchester KL: Some aspects of the kinetics of rat liver pyruvate carboxylase. Biochem J 1970, 120(1):79-93.
76. Wood HG, Davis JJ, Lochmuller H: The equilibria of reactions catalyzed by carboxytransphosphorylase, carboxykinase, and pyruvate carboxylase and the synthesis of phosphoendolpyruvate. The Journal of biological chemistry 1966, 241(23):5692-5704.

77. Wilson DF, Erecinska M, Schramm VL: Evaluation of the relationship between the intra- and extramitochondrial [ATP]/[ADP] ratios using phosphoenolpyruvate carboxykinase. The Journal of biological chemistry 1983, 258(17):10464-10473.
78. Titheradge MA, Picking RA, Haynes RC: Physiological Concentrations of 2-Oxoglutarate Regulate the Activity of Phosphoenolpyruvate Carboxykinase in Liver. Biochemical Journal 1992, 285:767-771.
79. Colombo G, Carlson GM, Lardy HA: Phosphoenolpyruvate Carboxykinase (Guanosine Triphosphate) from Rat-Liver Cytosol - Separation of Homogeneous Forms of Enzyme with High and Low Activity by Chromatography on Agarose-Hexane-Guanosine Triphosphate. Biochemistry 1978, 17(25):5321-5329.
80. Ballard FJ, Hanson RW: Phosphoenolpyruvate carboxykinase and pyruvate carboxylase in developing rat liver. Biochem J 1967, 104(3):866-871.
81. Jo JS, Ishihara N, Kikuchi G: Occurrence and Properties of 4 Forms of Phosphoenolpyruvate Carboxykinase in Chicken Liver. Archives of biochemistry and biophysics 1974, 160(1):246-254.
82. Johnson TA, Holyoak T: Increasing the Conformational Entropy of the Omega-Loop Lid Domain in Phosphoenolpyruvate Carboxykinase Impairs Catalysis and Decreases Catalytic Fidelity. Biochemistry 2010, 49(25):5176-5187.
83. Ballard FJ: Kinetic studies with cytosol and mitochondrial phosphoenolpyruvate carboxykinases. Biochem J 1970, 120(4):809-814.
84. Holyoak T, Nowak T: pH dependence of the reaction catalyzed by avian mitochondrial phosphoenolpyruvate carboxykinase. Biochemistry 2004, 43(22):7054-7065.
85. Palmieri F, Quagliar.E, Stipani I, Klingenb.M: Kinetic Study of Tricarboxylate Carrier in Rat-Liver Mitochondria. European Journal of Biochemistry 1972, 26(4):587-&.
86. Uyeda K, Furuya E, Luby LJ: The Effect of Natural and Synthetic D-Fructose 2,6-Bisphosphate on the Regulatory Kinetic-Properties of Liver and Muscle Phosphofructokinases. Journal of Biological Chemistry 1981, 256(16):8394-8399.
87. Van Schaftingen E, Jett MF, Hue L, Hers HG: Control of liver 6-phosphofructokinase by fructose 2,6-bisphosphate and other effectors. Proc Natl Acad Sci U S A 1981, 78(6):3483-3486.
88. Reinhart GD, Lardy HA: Rat liver phosphofructokinase: kinetic activity under near-physiological conditions. Biochemistry 1980, 19(7):1477-1484.
89. Sakakibara R, Kitajima S, Uyeda K: Differences in kinetic properties of phospho and dephospho forms of fructose-6-phosphate, 2-kinase and fructose 2,6-bisphosphatase. The Journal of biological chemistry 1984, 259(1):41-46.
90. Van Schaftingen E, Davies DR, Hers HG: Inactivation of phosphofructokinase 2 by cyclic AMP - dependent protein kinase. Biochem Biophys Res Commun 1981, 103(1):362-368.
91. Vanschaftingen E, Davies DR, Hers HG: Fructose-2,6-Bisphosphatase from Rat-Liver. European Journal of Biochemistry 1982, 124(1):143-149.
92. Cornell NW, Leadbetter M, Veech RL: Effects of free magnesium concentration and ionic strength on equilibrium constants for the glyceraldehyde phosphate dehydrogenase and phosphoglycerate kinase reactions. The Journal of biological chemistry 1979, 254(14):6522-6527.
93. Krietsch WK, Bucher T: 3-phosphoglycerate kinase from rabbit sceletal muscle and yeast. Eur J Biochem 1970, 17(3):568-580.
94. Fritz PJ, White EL: 3-Phosphoglycerate kinase from rat tissues. Further characterization and developmental studies. Biochemistry 1974, 13(3):444-449.

95. Clarke JB, Birch M, Britton HG: The equilibrium constant of the phosphoglyceromutase reaction. Biochem J 1974, 139(3):491-497.
96. Fundele R, Krietsch WK: Purification and properties of the phosphoglycerate mutase isozymes from the mouse. Comp Biochem Physiol B 1985, 81(4):965-968.
97. Middleton MC, Walker DG: Comparison of the properties of two forms of pyruvate kinase in rat liver and determination of their separate activities during development. Biochem J 1972, 127(4):721-731.
98. van Berkel TJ, Kruijt JK, Koster JF: Hormone-induced changes in pyruvate kinase. Effects of glucagon and starvation. Eur J Biochem 1977, 81(3):423-432.
99. Walker PR, Potter VR, Becker JE, Bonney RJ: Pyruvate-Kinase, Hexokinase, and Aldolase Isoenzymes in Rat-Liver Cells in Culture. In Vitro Cell Dev B 1972, 8(2):107-&.
100. Titheradge MA, Coore HG: Mitochondrial Pyruvate Carrier, Its Exchange Properties and Its Regulation by Glucagon. FEBS letters 1976, 63(1):45-50.
101. Halestrap AP: The mitochondrial pyruvate carrier. Kinetics and specificity for substrates and inhibitors. Biochem J 1975, 148(1):85-96.
102. Lee EW, Barriso JA, Pepe M, Snyder R: Purification and Properties of Liver Triose Phosphate Isomerase. Biochimica et biophysica acta 1971, 242(1):261-&.
103. Turnquis.Rl, Gillett TA, Hansen RG: Uridine-Diphosphate Glucose Pyrophosphorylase - Crystallization and Properties of Enzyme from Rabbit Liver and Species Comparisons. Journal of Biological Chemistry 1974, 249(23):7695-7700.
